# Beyond nitrogen: an oxygen-chemical-potential framework for defect-engineered TiO_2_ nanotube architectures in energy conversion and environmental catalysis

**DOI:** 10.1039/d6na00756b

**Published:** 2026-10-23

**Authors:** Hakimeh Ghazaie, Shahab Khameneh Asl, Shahin Khameneh Asl, Nurbek Rakhimov, Shohruh Sayfulloev, Sardorbek Saydaliev, Alisher Kahramonov, Utkirjon Sharopov

**Affiliations:** a University of Tabriz Tabriz Iran; b Karshi State Technical University Karshi Uzbekistan; c National University of Uzbekistan Named After Mirzo Ulugbek Tashkent Uzbekistan; d S.A. Azimov Physical-Technical Institute of Uzbekistan Academy of Sciences Tashkent Uzbekistan utkirstar@gmail.com utkirstar@uzsci.net

## Abstract

Nitrogen-doped titanium dioxide (TiO_2_) nanotube arrays (N-TNTs) have become a reference platform for solar energy conversion and electrochemical storage, yet the field remains organised around a material-centric narrative in which nitrogen is treated as the primary functional agent and each application is optimised in isolation. Here we argue for a different reading. Drawing together evidence from lithium-ion storage, supercapacitive charge storage, photoelectrochemical water splitting and visible-light photocatalysis, we show that the performance-determining quantity in these systems is not the nitrogen content itself but the coupled population of shallow oxygen vacancies (V_o_) and Ti^3+^ centres—a single defect state whose equilibrium is set, during synthesis and post-treatment, by the oxygen chemical potential (*µ*_o_). In this view nitrogen acts chiefly as a thermodynamic stabiliser that pins this defect state and shifts its formation window, rather than as an independent visible-light ‘switch’. Re-examining the literature through this lens reveals that the four applications are not separate optimisation problems but different projections of the same underlying defect equilibrium, and that their apparently disparate optimal conditions collapse onto a common design window in the *µ*_o_-defect-density plane. We formalise this as a defect-state design map, propose a compact and transferable defect-state descriptor together with a minimum defect-reporting protocol to make results comparable across laboratories, and outline how *operando* defect metrology and closed-loop, machine-learning-guided synthesis can convert empirical doping into predictive defect-equilibrium engineering. This reframing offers a unifying and testable basis for designing N-TNT architectures—and, more broadly, defective oxide nanomaterials—for robust, scalable energy and environmental devices.

## Abstract 2

Nitrogen acts as a stabiliser rather than the active ingredient: the oxygen chemical potential sets one shallow V_o_/Ti^3+^ defect state, of which lithium storage, supercapacitance, photoelectrochemistry and photocatalysis are four projections.

## Graphical abstract

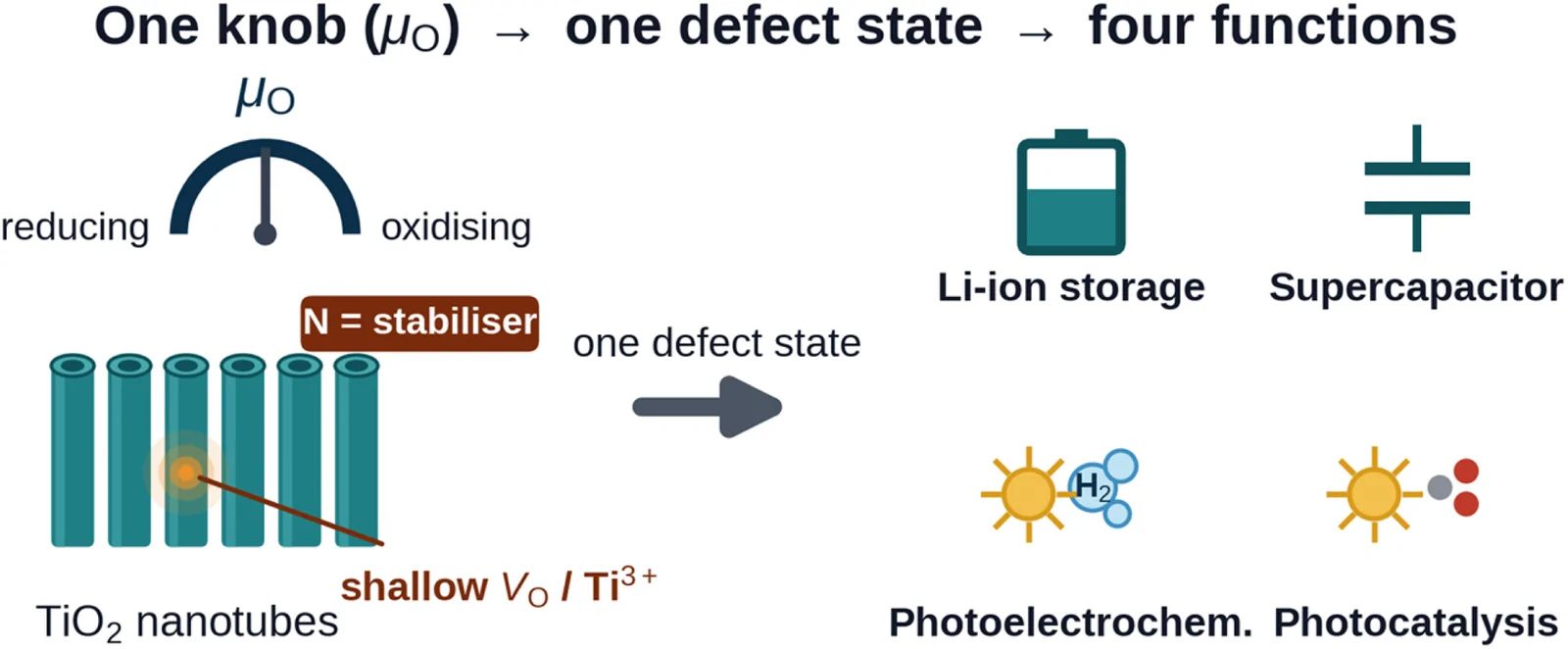

## 1. Introduction

Titanium dioxide (TiO_2_) is widely recognized as a robust and economically viable semiconductor for energy conversion and environmental technologies due to its chemical stability, low toxicity, and favorable band-edge positions.^1,2^ Nevertheless, its large bandgap (∼3.2 eV for anatase) restricts optical absorption predominantly to the ultraviolet region, while rapid electron–hole recombination significantly limits charge utilization efficiency.^3^ These intrinsic limitations have motivated extensive engineering strategies to tailor the electronic structure, defect chemistry, and interfacial properties of TiO_2_ in order to enhance visible-light responsiveness and improve carrier-separation efficiency for practical device applications.^4,5^

TiO_2_ nanotubes (TNTs) offer a one-dimensional, vertically aligned architecture that combines high surface area with direct electron transport pathways, making them particularly attractive for electrochemical and photoelectrochemical device integration.^6^ These nanotube arrays are most commonly fabricated *via* electrochemical anodization of titanium, a scalable technique that provides deterministic control over geometric parameters-including tube length, pore diameter, wall thickness, and array ordering-through adjustment of applied voltage, electrolyte composition, temperature, and anodization time.^7–9^ Subsequent thermal treatment governs crystallinity and phase composition, thereby directly influencing charge transport, interfacial kinetics, and overall electrochemical or photocatalytic performance.^10^

The intrinsic electronic limitations of TNTs can be strategically addressed through controlled doping that modulates band structure and defect chemistry. Among various approaches, nitrogen incorporation has emerged as a particularly effective route for engineering TiO_2_, as it introduces impurity states within the bandgap, adjusts Fermi-level positioning, and modifies band-edge alignment, thereby improving visible-light absorption and charge transport behavior.^11,12^ Nitrogen may occupy substitutional or interstitial sites, each configuration leading to distinct electronic and defect-related effects. Although other dopants-including non-metals,^13^ transition metals,^14^ and rare-earth elements^15^-have been investigated in TiO_2_ systems, this review concentrates specifically on nitrogen doping as a controllable and application-driven strategy for tailoring TNT architectures.

It is important to distinguish between TiO_2_ polymorphs (anatase, rutile, and brookite) and titanate-derived compounds such as Li_4_Ti_5_O_12_, which exhibit fundamentally different crystal structures and electrochemical behaviors.^16^ While titanates are widely studied as intercalation anodes in lithium-ion batteries, the present review is confined to N-doped TiO_2_ nanotubes (N-TNTs) architectures derived from anatase/rutile phases, with titanates mentioned only for contextual comparison in energy-storage discussions.

Nitrogen doping has been widely reported to introduce localized electronic states near the valence-band edge of TiO_2_, resulting in band-edge modulation and enhanced visible-light activity.^17^ Beyond optical effects, nitrogen incorporation modifies defect chemistry, carrier mobility, and recombination dynamics-parameters that are directly relevant to device-level performance. Such doping-induced electronic tuning plays a critical role in optimizing electrodes for high-rate lithium-ion storage and photoelectrochemical applications. In energy-storage systems, N-TNT arrays have been investigated as advanced electrode architectures for lithium-ion batteries, sodium-ion batteries, and supercapacitors, where improved electronic conductivity and stabilized defect structures contribute to enhanced rate capability and cycling stability.^18–20^ The intrinsic one-dimensional geometry of the nanotubes further promotes directional ion transport and reduced internal resistance, supporting efficient electrochemical operation.

In environmental applications, N-TNT arrays function as visible-light-responsive photocatalysts for the degradation of organic contaminants, including dyes and pharmaceutical residues.^21,22^ Nitrogen incorporation extends optical absorption into the visible region and modulates surface redox activity, while the ordered nanotubular architecture enhances charge separation and interfacial reaction kinetics. These combined effects enable improved degradation efficiency and position N-TNTs as promising platforms for sustainable environmental remediation technologies.

Theoretical modeling, particularly density functional theory (DFT), has contributed to understanding how nitrogen incorporation modifies the electronic structure and defect energetics of TiO_2_. Computational studies indicate that substitutional nitrogen can promote valence-band modulation and stabilize oxygen-vacancy-related donor states, whereas excessive interstitial nitrogen tends to introduce mid-gap trap states associated with recombination. When interpreted alongside spectroscopic analyses (*e.g.*, XPS, UV-vis, PL, EPR), these insights provide a mechanistic foundation for defect-controlled engineering of N-TNTs. In parallel, advances in synthesis and post-treatment strategies have enabled more precise control over dopant distribution and defect density at the nanoscale.^23^

Despite notable advances, several engineering challenges continue to limit the practical deployment of N-TNT architectures. Achieving precise control over dopant concentration, spatial distribution, and chemical configuration remains critical to prevent phase instability, excessive defect clustering, or performance degradation. Furthermore, scalable fabrication routes capable of producing uniform and reproducible N-doped nanotube arrays in industrially relevant quantities are still insufficiently developed.^24^ Overcoming these barriers requires a rigorous understanding of the process–structure–property relationships that govern electronic transport, interfacial kinetics, and long-term device stability.

In response to the growing demand for sustainable and high-performance materials, recent studies (2023–2025) have reported improved nitrogen-incorporation strategies and the development of hybrid N-TNT architectures with enhanced structural stability and electrochemical efficiency.^25,26^ These advances emphasize controlled defect engineering, optimized nanotube geometry, and improved interfacial integration, further expanding the applicability of N-TNTs in energy-storage and photoelectrochemical systems.

### 1.1 Why this review is needed now: three unresolved bottlenecks

Although the N-TNT literature is large and rapidly growing, its collective value is limited by three structural problems that recur across otherwise excellent individual studies. We set them out explicitly here because they define both the timing and the purpose of this review.

The first bottleneck is fragmented empirical optimisation. Nitrogen-doped nanotubes are optimised separately for lithium-ion storage, supercapacitance, photoelectrochemical water splitting and photocatalysis, and the ‘optimal’ synthesis conditions are rediscovered independently within each community. The same material class is reported with incompatible optima: annealing temperatures that maximise anatase crystallinity for photoanodes^27^ are treated as excessive for supercapacitor electrodes that rely on Ti^3+^-rich surfaces,^19,28^ while nitrogen loadings that improve visible-light photocatalysis^21,22^ are reported as detrimental to rate capability in Li-ion anodes.^29,30^ Because these optima are expressed in application-specific metrics rather than in a common material descriptor, they do not transfer, and each new application restarts the search.

The second bottleneck is inconsistent defect reporting. Across the studies compiled in Tables 7–10, a large fraction report only the total nitrogen content (at%) without separating substitutional (N_sub_) from interstitial (N_int_) configurations, provide no quantitative measure of oxygen-vacancy or Ti^3+^ density, and omit the post-treatment atmosphere, oxygen partial pressure, temperature ramp or dwell time—precisely the variables that fix the defect state. Geometric parameters are frequently given as single ‘representative’ values rather than distributions. As a result, two samples described as ‘N-doped TNTs, 2 at% N’ can differ by an order of magnitude in donor density and cannot be placed on any common scale; genuine trends are buried under unreported process variables.

The third bottleneck is the contradictory functional interpretation of nitrogen itself. One body of work attributes performance gains to band-gap narrowing by N 2p states above the valence band;^12,17^ a second attributes them to nitrogen-induced oxygen vacancies and Ti^3+^ donors;^31,32^ a third reports that ‘excess’ nitrogen degrades performance through mid-gap trap formation and recombination.^33,34^ These interpretations are usually presented as competing, yet they are reconciled once it is recognised that nitrogen and oxygen vacancies are thermodynamically coupled—substitutional nitrogen lowers the formation energy of V_o_^31,35^—so that the observable outcome depends on the oxygen chemical potential (*µ*_o_) imposed during processing rather than on nitrogen content alone.

The oxygen-chemical-potential framework developed in this review addresses each of these demands directly. Treating *µ*_o_ as the single governing variable, and the coupled shallow-V_o_/Ti^3+^ population as the defect state it controls, allows the optima reported for different applications to be expressed on one axis and therefore transferred between them (bottleneck 1). A minimum defect-reporting protocol, formalised in Section 3.7, specifies the small set of measurements and process variables needed to place any sample on that axis and thus renders results from different laboratories comparable (bottleneck 2). Finally, interpreting nitrogen as a thermodynamic stabiliser that pins and shifts the accessible defect state—rather than as the primary functional species—reconciles the conflicting accounts of its role (bottleneck 3): band-gap narrowing, donor generation and trap formation are not rival mechanisms but different regions of the same *µ*_o_-controlled defect landscape.

### 1.2 Scope and novelty of this review

Several authoritative reviews cover TiO_2_ nanotubes and nitrogen-doped TiO_2_, and the present work builds on them. The formation, properties and applications of anodic TNT arrays have been comprehensively reviewed by Macak *et al.*,^5^ Roy, Berger and Schmuki^6^ and Lee, Mazare and Schmuki;^36^ nitrogen-doped TiO_2_ photocatalysts by Asahi *et al.*;^34^ defective (‘black’) TiO_2_ by Chen, Liu and Huang;^32^ and application-specific N-TNT systems by Fu and Mo^37^ and Hou, Aitola and Lund.^38^ As Table 1 makes explicit, all of these are organised around nitrogen (or another dopant) as the active agent and treat each application within its own frame of reference; none identifies a single processing variable that governs the defect state across applications, none proposes a quantitative, transferable defect-state descriptor, and none specifies a reporting protocol that would make the resulting data comparable.

**Table 1:** Comparison of the present review with representative existing reviews on TiO_2_ nanotubes and nitrogen-doped TiO_2_. ✓ = treated explicitly; (✓) = partially/implicitly; ✗ = not addressed

Review (ref.)	Primary focus	Applications covered	Role assigned to nitrogen	V_o_/Ti^3+^ as coupled state	*µ*_o_ as master variable	Defect-state descriptor	Reporting protocol	Design map
Macak *et al.*, 2007 (ref. 5)	Self-organised anodic TNTs: formation & properties	Photocatalysis, sensing, biomedical	Not central	✗	✗	✗	✗	✗
Roy, Berger & Schmuki, 2011 (ref. 6)	TNT synthesis & applications	DSSC, photocatalysis, biomedical, batteries	One dopant among many	✗	✗	✗	✗	✗
Lee, Mazare & Schmuki, 2014 (ref. 36)	1D TiO_2_ nanotubes (comprehensive)	Broad (energy, environment, biomedical)	One dopant among many	(✓)	✗	✗	✗	✗
Asahi *et al.*, 2014 (ref. 34)	N-doped TiO_2_ photocatalysis	Photocatalysis	Active agent (band-gap narrowing)	(✓) N–V_o_ interplay discussed	✗	✗	✗	✗
Chen, Liu & Huang, 2015 (ref. 32)	Black/defective TiO_2_	Photocatalysis, energy conversion	Secondary to V_o_/Ti^3+^	✓ (defects central)	✗	✗	✗	✗
Fu & Mo, 2018 (ref. 37)	Self-organised TNTs: modifications & biomedical use	Biomedical, photocatalysis	Minor	✗	✗	✗	✗	✗
Hou, Aitola & Lund, 2021 (ref. 38)	TNTs for dye-sensitised solar cells	DSSC	Dopant for band-edge tuning	✗	✗	✗	✗	✗
This review	N-TNT defect equilibrium across applications	LIB, SC, PEC, photocatalysis, DSSC	Thermodynamic stabiliser of the V_o_/Ti^3+^ state	✓	✓ (*µ*_o_)	✓ (Section 3.7, Conclusions)	✓ (Section 3.7)	✓ (Fig. 13)

The unique contributions of this review are therefore fourfold. (i) It identifies the oxygen chemical potential *µ*_o_, set by growth, annealing and nitridation, as the master variable of N-TNT defect chemistry. (ii) It reinterprets nitrogen as a thermodynamic stabiliser of the coupled shallow-V_o_/Ti^3+^ state rather than as the primary functional species. (iii) It shows that lithium-ion storage, supercapacitance, photoelectrochemistry and photocatalysis are projections of one defect equilibrium whose optima converge in a common ‘synergy window’, rendered as a defect-state design map (Fig. 13). (iv) It makes the framework operational through a compact defect-state descriptor and a minimum reporting protocol (Section 3.7), complemented by a survey of *operando* probes able to follow the defect equilibrium during operation (Section 7.4). Fig. 1 contrasts this organisation with the conventional nitrogen-centric, application-by-application structure of the existing literature and provides a roadmap of the review.

**Fig. 1:**
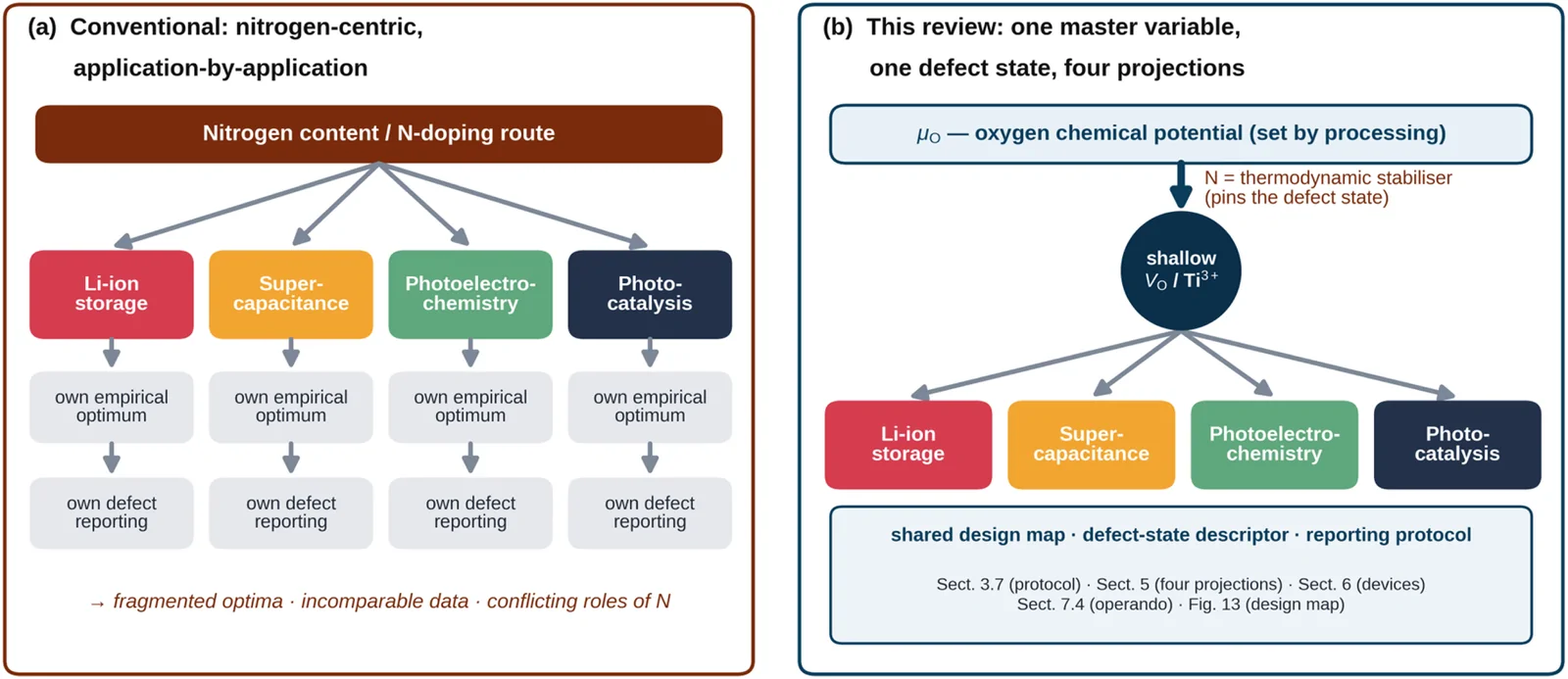
Organisation of the field and of this review. (a) Conventional, nitrogen-centric organisation: each application is optimised independently against its own empirical optimum and reports defects in its own way, producing fragmented optima, incomparable data and conflicting interpretations of the role of nitrogen. (b) The framework adopted here: the oxygen chemical potential *µ*_o_ (set by processing) governs a single shallow-V_o_/Ti^3+^ defect state, which nitrogen stabilises, and the four application classes are treated as projections of that one state. A shared design map, a quantitative defect-state descriptor and a minimum reporting protocol (Sections 3.7 and 7.4) connect the projections; the boxes indicate where each element is developed in the review.

### 1.3 Aims and organisation

The aim of this review is to provide a comprehensive engineering-oriented analysis of nitrogen-doped TiO_2_ nanotube architectures and their role in advanced energy-storage and photoelectrochemical systems. Specifically, this work focuses on establishing a process–structure–performance framework that links synthesis conditions, defect chemistry, and nanotube geometry to measurable device-level performance.

In this context, the present review provides a focused and application-driven assessment of N-TNTs architectures, emphasizing structural engineering principles and their implications for energy-storage and photoelectrochemical device performance. Although several recent reviews have examined TiO_2_-based materials, nanotube morphologies, or broadly doped TiO_2_ systems, these studies typically encompass multiple dopants and application domains without establishing a clear process–structure–performance framework specific to nitrogen-doped anodic TNTs.^25,39^ Here, we aim to consolidate existing knowledge into an engineering-oriented perspective that links controlled nitrogen incorporation and nanotube architecture to measurable functional metrics. Against this backdrop, the present review moves beyond a descriptive survey by establishing a structured, engineering-oriented framework for N-TNT architectures.

First, we organize synthesis and post-treatment strategies within an explicit process–structure–performance model that links anodization parameters, thermal treatment conditions, and nitrogen incorporation pathways to defect chemistry, electronic structure evolution, and measurable device metrics.

Second, through comparative cross-application analysis encompassing lithium-ion storage, supercapacitive behavior, photocatalysis, and photoelectrochemical conversion, we identify recurring design windows-defined by nanotube geometry, phase composition, nitrogen configuration, and charge-transfer characteristics-that consistently correlate with enhanced functional performance.

Third, we integrate mechanistic insights from density functional theory with spectroscopic and electrochemical evidence to rationalize the synergistic roles of nitrogen, Ti^3+^ centers, and oxygen vacancies in governing carrier transport and recombination dynamics. Collectively, this integrated and design-driven perspective provides a predictive roadmap for the scalable and durable engineering of N-TNT systems while clarifying key challenges for future device-level implementation.

A central argument of this review is conceptual rather than incremental. The prevailing literature frames nitrogen as the active ingredient of N-TNTs and evaluates each device application against its own optimum. We instead propose that these systems are governed by a single, tunable defect equilibrium—the coupled concentration of substitutional nitrogen (N_sub_, replacing lattice oxygen), shallow oxygen vacancies (V_o_) and Ti^3+^ centres—whose state is controlled by the oxygen chemical potential (*µ*_o_) imposed during growth, annealing and nitridation. Within this framework nitrogen is best understood as a stabiliser that pins and shifts the accessible defect state, whereas *µ*_o_ is the master variable that the experimentalist actually sets. The practical consequence is that lithium-ion storage, supercapacitance, photoelectrochemistry and photocatalysis become different projections of one defect state, and their design rules can be read from a common map rather than rediscovered application by application. We adopt this lens throughout the sections that follow and, in the concluding perspective, render it operational as a design map, a quantitative descriptor and a reporting protocol.

## 2. Fundamentals of TiO_2_ nanotubes

TiO_2_ exists primarily in anatase and rutile polymorphs, which exhibit distinct electronic structures and transport characteristics that directly influence device performance.^1,3^ Anatase is generally favored in photocatalytic and electrochemical systems due to its higher carrier mobility and slower recombination kinetics, whereas rutile offers superior thermodynamic stability but typically lower photoactivity. Controlled phase composition therefore represents a critical engineering parameter in TNT design.

As-formed TNTs are amorphous and require post-annealing to achieve crystalline phases. Thermal treatment between 350–500 °C typically yields anatase-dominant structures suitable for energy and photoelectrochemical applications, whereas higher temperatures (>600 °C) promote rutile formation and modify charge-transport characteristics.^27^

## 3. Structural engineering strategies for TiO_2_ nanotubes

### 3.1 Structural engineering-geometric design windows (*D*, *t*, *L*, porosity)

The performance of N-TNT arrays is governed by the coordinated optimization of three interdependent design parameters: (i) nanotube geometry (pore diameter, wall thickness, length, and array density), (ii) nitrogen chemical configuration and spatial distribution (substitutional *versus* interstitial states and depth profile), and (iii) phase and defect landscape (anatase-rutile balance and Ti^3+^/oxygen-vacancy populations). Effective structural engineering therefore requires linking controllable processing variables-such as anodization parameters and post-treatment conditions-to geometric attributes, dopant configuration, and defect chemistry, and subsequently to optoelectronic properties and measurable device metrics. In this section, we synthesize recurring process–structure–performance relationships reported for TNT systems in photocatalytic and photoelectrochemical applications, establishing practical design windows for high-performance N-TNT architectures.^39^

### 3.2 Nitrogen incorporation & spatial distribution (N_sub_*vs.* N_int_)

Nitrogen incorporation into TNT arrays can be achieved either *in situ* during growth or *via* post-synthetic treatments such as NH_3_/N_2_ annealing, plasma nitriding, or ion implantation. Post-treatment strategies are particularly advantageous for nanotube systems because they decouple structural formation from dopant chemistry, enabling independent control over nitrogen configuration and depth distribution. Moderate NH_3_/N_2_ annealing (400–500 °C) or controlled plasma nitriding has been shown to incorporate nitrogen while preserving nanotube morphology, simultaneously enhancing visible-light absorption and photocurrent response.^40–42^

X-ray photoelectron spectroscopy (XPS) is the primary tool for distinguishing nitrogen configurations: substitutional nitrogen (N_sub_), replacing lattice oxygen, typically appears at N 1s ≈ 396–397 eV, whereas interstitial or molecular nitrogen species (N_int_) are detected at ≈399–401 eV.^43–45^ Because peak overlap with surface nitride-like species can occur, reliable assignment requires complementary analysis of Ti 2p spectra and, where possible, depth-resolved techniques such as angle-resolved XPS, ToF-SIMS, or EPR.

From a structural-engineering standpoint, an N_sub_-dominant configuration combined with controlled oxygen-vacancy (V_o_) density represents a favorable design regime. Substitutional nitrogen promotes N 2p-O 2p hybridization near the valence-band edge and can reduce V_o_ formation energy, thereby enhancing donor density and charge transport when defect populations remain shallow. In contrast, excessive interstitial nitrogen introduces mid-gap trap states that accelerate recombination. Consequently, processing parameters-including NH_3_/N_2_ flow rate, treatment temperature and duration, or plasma power and duty cycle-must be optimized to maximize N_sub_ while preventing excessive defect clustering.^31,46^

### 3.3 Phase & defect engineering (anatase/rutile, Ti^3+^/V_o_)

Phase composition and defect engineering are as critical as nitrogen configuration in determining the performance of N-TNT arrays. Anatase-dominant architectures generally provide longer carrier lifetimes and higher mobility, whereas the introduction of a controlled rutile fraction can generate interfacial band offsets that facilitate spatial charge separation along the nanotube walls. Experimental kinetic analyses and time-resolved photoluminescence studies at anatase-rutile junctions consistently demonstrate reduced recombination when mixed-phase ratios are judiciously optimized.^16^

The electronic interaction between anatase and rutile depends on their relative band alignment. In favorable staggered (type-II-like) configurations, electrons and holes preferentially localize in different phases, thereby suppressing interfacial recombination. However, band alignment can vary depending on surface termination, defect density, and measurement conditions, indicating that mixed-phase advantages are not universal but contingent on controlled interfacial continuity and quantified phase fractions.^47^ Accordingly, Rietveld-refined XRD analysis is essential to accurately determine polymorph balance after nitridation or thermal treatments.

Defect engineering-particularly the regulation of Ti^3+^ centers and oxygen vacancies (V_o_)-plays a decisive role in controlling donor density, band bending, and trap-assisted recombination in N-TNT arrays. A shallow-defect “design window” is generally observed: moderate Ti^3+^/V_o_ concentrations enhance electronic conductivity and beneficially widen space-charge regions, as evidenced by Mott–Schottky analysis and electrochemical impedance spectroscopy (EIS). In contrast, excessive defect densities introduce deep mid-gap trap states that accelerate recombination, shorten carrier lifetimes, and suppress photocurrent generation.^48–50^ Studies on defect-engineered or partially reduced (“black”) TiO_2_ demonstrate that controlled self-doping improves visible-light absorption and activity, whereas over-reduction or aggressive nitridation leads to trap proliferation and performance degradation.

Processing sequence is therefore critical. A practical engineering strategy involves initial crystallization of the nanotube scaffold in air or O_2_ to establish anatase texture and grain structure, followed by moderated nitridation (NH_3_/N_2_ annealing or low-temperature plasma treatment) to introduce nitrogen while preserving crystallinity and maintaining shallow defect populations. Surface-sensitive spectroscopies (*e.g.*, XPS, NEXAFS) indicate that severe NH_3_ treatments can generate molecular nitrogen species or nitride-like surface phases, underscoring the need for controlled conditions; plasma nitriding at reduced temperatures offers improved tunability of dopant depth and defect density.^51^

Reliable structure–defect–performance correlations require a minimum characterization framework. Phase fractions and crystallite sizes should be quantified by Rietveld-refined XRD; nitrogen configuration and Ti^3+^/V_o_ states verified by XPS and EPR; recombination dynamics assessed *via* TRPL or steady-state PL; and charge-transport properties evaluated using EIS and Mott–Schottky analysis under standardized illumination and electrolyte conditions. Where mixed-phase mechanisms are invoked, time-resolved probes are necessary to substantiate enhanced interfacial charge separation.^52^

From a design perspective, anatase-dominant nanotubes with a modest rutile fraction, N_sub_-dominant chemistry, and controlled shallow V_o_ concentrations consistently define a favorable operating regime. This regime is typically characterized by measurable Ti^3+^ signatures in EPR without excessive trap formation, moderate increases in donor density (N_D_), preserved carrier lifetimes, and sustained performance gains under standardized testing protocols.^16,53^

### 3.4 Structural engineering-process → structure map for N-doped TiO_2_ nanotubes

A structured process-structure map provides a practical framework linking controllable fabrication parameters to geometric attributes, phase composition, and nitrogen chemistry in N-TNT arrays. Three primary processing stages govern final architecture: (i) anodization conditions, which determine pore diameter, wall thickness, tube length, and array ordering; (ii) initial air/O_2_ annealing, which establishes anatase texture and crystallite size; and (iii) subsequent NH_3_/N_2_ annealing or plasma nitridation, which regulates the N_sub_/N_int_ ratio and oxygen-vacancy depth and density. These dependencies can be visualized in a two-panel map: the first panel correlates anodization variables (applied voltage, water content, fluoride concentration, and time) with geometric outputs (*D*, *t*, *L*, ordering), while the second links post-treatment temperature and nitridation severity to nitrogen configuration and defect profiles. Practical operating windows for reproducible fabrication and performance targeting are defined within these parameter spaces.^6,37^

Within the anodization domain, increasing applied voltage generally enlarges pore diameter, whereas higher fluoride concentration and water content alter the growth-dissolution equilibrium, promoting thinner walls and higher porosity. Anodization time increases tube length until transport limitations impose a plateau. Electrolyte aging further modifies conductivity and growth kinetics, often resulting in shorter nanotubes.^6,54^ These coupled effects define geometry trade-offs that must be optimized according to targeted electrochemical or photoelectrochemical transport requirements.

Fig. 2 shows how the water and fluoride contents of the electrolyte set the pore geometry of the as-anodized arrays on which the subsequent post-treatment acts. The post-treatment design space governing nitrogen configuration and defect evolution in N-TNTs can be summarized as follows. Moderate annealing temperatures combined with controlled NH_3_ flow rates or mild plasma exposure favor substitutional nitrogen incorporation (N 1s ≈ 396–397 eV) together with shallow oxygen-vacancy populations, typically accompanied by modest Ti^3+^ signatures. Increasing treatment severity-*via* higher temperature, extended duration, elevated NH_3_ flow, or stronger plasma duty-shifts spectral intensity toward interstitial configurations (≈399–401 eV), increases trap depth and density, enhances charge-transfer resistance (*R*_ct_), quenches PL/TRPL lifetimes, and ultimately degrades photocurrent density and IPCE.^40,55,56^ Together with the geometric window illustrated in Fig. 2, these trends therefore delineate a practical engineering window in which N_sub_-dominant chemistry and shallow V_o_ concentrations are achieved while preserving crystallinity and carrier transport.

**Fig. 2:**
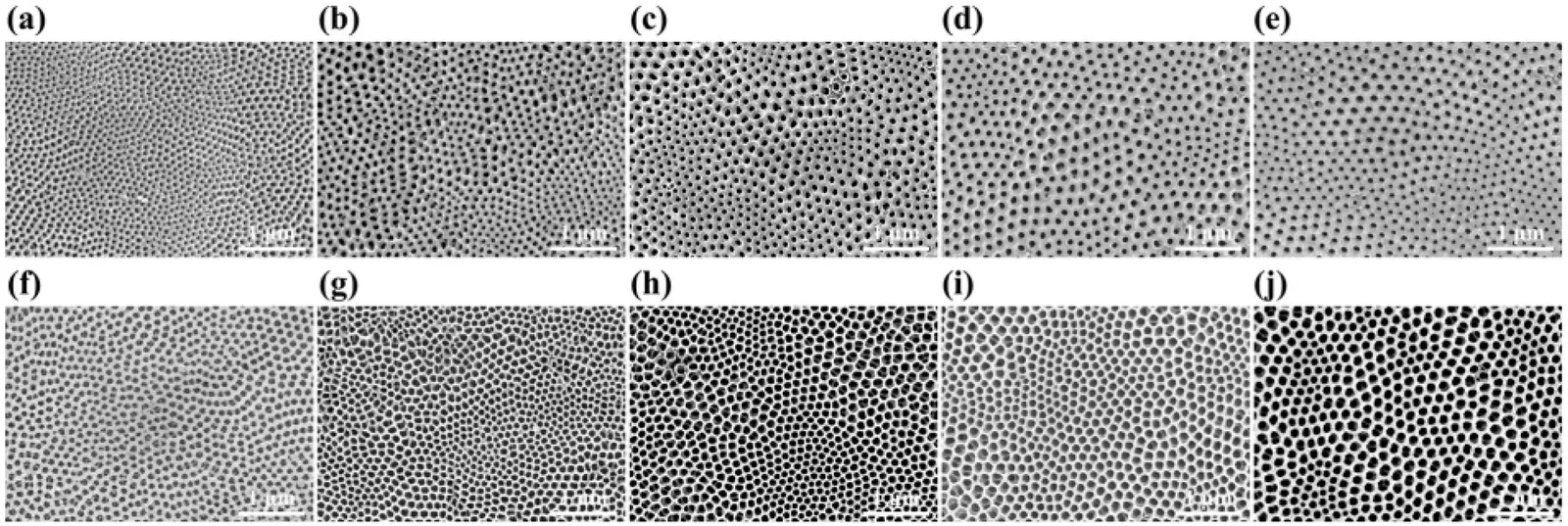
Top-view SEM images of TiO_2_ nanotube arrays anodized at 60 V for 1 h in ethylene glycol containing (a–e) 2, 4, 6, 8 and 10 vol% water with 0.2 wt% NH_4_F and (f–j) 2, 4, 6, 8 and 10 vol% water with 0.4 wt% NH_4_F, showing the dependence of pore geometry on electrolyte composition. Reproduced from ref. 56 (J. Kim, B. Kim, C. Oh, J. Ryu, H. Kim, E. Park, K. No and S. Hong, effects of NH_4_F and distilled water on structure of pores in TiO_2_ nanotube arrays, *Scientific Reports*, 2018, **8**, DOI: https://doi.org/10.1038/s41598-018-30668-3) under the terms of the Creative Commons Attribution 4.0 licence (CC BY 4.0), copyright 2018 The Authors.

### 3.5 Structure–property–performance matrix

To operationalize the structural-engineering framework developed in Sections 3.1–3.4, Table 2 establishes an explicit structure–property–performance (SPP) matrix linking geometric parameters, phase composition, nitrogen configuration, and defect populations to measurable optoelectronic properties and device-level metrics. In anodized TNT arrays, processing variables-including applied voltage, water and fluoride concentration, and anodization time-deterministically define pore diameter, wall thickness, tube length, and ordering. These geometric design windows directly influence space-charge width, series resistance, and electrolyte accessibility, thereby impacting charge transport and interfacial kinetics.^6,56^

**Table 2:** Quantitative structure–property–performance (SPP) design matrix for engineering N-TNT arrays

Structure descriptor	Typical window/level	Mechanistic effect	Performance implication
Pore diameter	50–150 nm	Depletion width and optical coupling depend on diameter; very small diameters raise surface recombination; very large diameters reduce active area/field overlap	Intermediate (∼80–120 nm) frequently yields higher IPCE and *J*_ph_^56^
Wall thickness	10–30 nm	Ultra-thin walls amplify surface states unless passivated; thick walls add series resistance	An intermediate range around 15–25 nm balances transport and stability; ABPE and IPCE improve in this window^6^
Tube length	2–8 µm	Surface area increases with length, but transport losses appear beyond a saturation length	Around 3–6 µm, *J*_ph_ approaches a plateau^6^
Array ordering/porosity	Ordered, medium-to-high porosity	Determines electrolyte access and space-charge overlap	Ordered arrays with medium porosity aid mass transport; excessive porosity promotes coalescence and recombination^56^
Phase fraction (anatase/rutile)	Anatase-dominant with about 10–30% rutile	Interfacial offsets in mixed phases facilitate charge separation; excessive rutile shortens carrier lifetimes	Anatase-rich mixed phases frequently enhance IPCE and *J*_ph_^57,58^
N configuration	N_sub_ much greater than N_int_	N_sub_ produces valence-band uplift and shallow donor-like states; N_int_ introduces mid-gap traps	N_sub_-dominant chemistry improves visible response and transport; N_int_-rich chemistry degrades performance^31^
Ti^3+^/oxygen-vacancy depth and density	Shallow defects with moderate density	Donor density increases and flat-band potentials shift; deep traps shorten lifetimes	A moderate level (“sweet spot”) lowers *R*_ct_ and raises *J*_ph_; excessive reduction lowers performance^40^
Post-treatment severity	Moderate annealing temperature and NH_3_ flow/time; mild plasma if used	Controls the N_sub_/N_int_ ratio and vacancy depth	Moderate severity preserves crystallinity and favors N_sub_; aggressive treatments cause trap proliferation (see Fig. 13)^40^
Surface co-catalyst (*e.g.*, CoPi, NiFeO_*x*_, MXene, rGO)	Trace to near-monolayer, uniform coverage	Lowers *R*_ct_ and improves interfacial kinetics; excessive loading blocks light	Proper loadings increase ABPE and H_2_ rate; overloading is detrimental^6^
Ultrathin passivation (ALD Al_2_O_3_ or TiO_2_)	1–3 nm conformal coating	Reduces surface recombination with minimal band-edge shifts	IPCE and *J*_ph_ improve when mass transport is not limiting^6^

From a chemical standpoint, substitutional nitrogen (N_sub_), identified by N 1s components near ∼396–397 eV, promotes N 2p-O 2p hybridization and is typically associated with shallow oxygen-vacancy populations that enhance donor density without generating deep trap states. In contrast, interstitial or molecular nitrogen species (N_int_, ∼399–401 eV) tend to introduce mid-gap levels that accelerate recombination. Accordingly, post-treatment protocols (NH_3_/N_2_ annealing or plasma nitriding) are engineered to favor N_sub_ incorporation while constraining V_o_ to shallow concentrations.^31,40^

Phase composition further modulates carrier dynamics. Anatase-dominant nanotubes generally sustain longer carrier lifetimes and higher mobility, whereas the introduction of a controlled rutile fraction can create interfacial band offsets that facilitate charge separation. Excessive rutile content, however, may compromise transport continuity. Reliable quantification of phase fractions *via* Rietveld-refined XRD is therefore recommended whenever annealing or nitridation treatments are applied.^57,58^

When optimized geometric parameters (*D*/*t*/*L*), an anatase-rich mixed phase, and N_sub_-dominant chemistry with shallow V_o_ populations converge, the resulting architecture exhibits enhanced interfacial charge transfer and reduced recombination losses. This coordinated alignment is reflected in improved incident photon-to-current efficiency (IPCE), photocurrent density (*J*_ph_), applied bias photon-to-current efficiency (ABPE), hydrogen evolution rates, and pollutant-degradation kinetics under standardized testing conditions.^40,56^

### 3.6 Design rules & reporting checklist

Building upon Sections 2 and 3, we consolidate practical design rules and reporting standards to ensure that structure–property relationships in anodic TNTs are reproducible and quantitatively comparable across laboratories. Rather than prescribing fixed parameter values, the emphasis is placed on clarifying the cause-effect chain linking processing conditions to morphology, defect chemistry, and device performance.

From a fabrication standpoint, anodization in ethylene-glycol-based electrolytes is governed by field-assisted oxidation and fluoride-mediated dissolution. Water content and fluoride concentration jointly control barrier-layer formation, pore nucleation, tube ordering, and final length through their influence on dissolution kinetics and defect generation. Reproducible optimization therefore requires explicit reporting of voltage trajectory (including ramp and plateau stages), current–time (*I*–*t*) transients, electrolyte composition and purity, bath temperature and aging, electrode geometry, cell configuration, and full electrical traces, as these parameters define the growth regime and resulting geometry.^6,31,59^

Post-growth annealing establishes crystallinity and phase architecture. When mixed anatase-rutile systems are engineered to promote interfacial charge separation, phase fractions must be quantitatively determined (*e.g.*, *via* Rietveld refinement), and interfacial continuity should be substantiated rather than assumed. XRD acquisition parameters and complementary Raman analysis should be disclosed to anchor electronic interpretations in measured structure.^47,57^

Nitrogen incorporation is treated as controlled defect-chemistry engineering. Substitutional and interstitial nitrogen states exhibit distinct electronic effects and different coupling tendencies with oxygen vacancies. Because nitrogen can reduce V_o_ formation energy, visible-light enhancement may coincide with increased recombination if deep traps proliferate. A rational processing sequence therefore involves stabilizing the oxide phase prior to nitridation, followed by controlled NH_3_/N_2_ annealing or plasma treatment to favor substitutional incorporation (N 1s ≈ 396–397 eV) over interstitial species (≈399–401 eV), supported by transparent XPS fitting protocols and quantified N/Ti ratios.^15,31,40,46^

Plasma nitriding offers improved temporal control and reduced thermal budgets; however, instrument configuration, fitting models, uncertainty bounds, and raw spectral data should be reported to maintain analytical rigor. Tracking N 1s components alongside Ti^3+^/V_o_ signatures enables confinement within a defect-beneficial regime.^40^

Finally, structure–performance correlations should rely on statistical robustness rather than representative micrographs. Geometric parameters (*D*, *t*, *L*) should be reported as distributions (mean ± SD, *n* ≥ 50) with disclosed image-analysis methodology. Optical and (photo)electrochemical performance metrics-including photon-normalized efficiencies (AQE/APCE), spectral irradiance, illuminated area, catalyst loading, reference electrode and potential scale, electrolyte composition, and stability criteria-must be explicitly stated, as these boundary conditions govern comparability.^6,31,59^

Collectively, adherence to quantified process control, transparent defect characterization, and standardized performance reporting transforms TNT research from descriptive experimentation into cumulative engineering science.

### 3.7 A minimum defect-reporting protocol for N-TNT studies

The reporting checklist of Section 3.6 is here consolidated into an explicit minimum protocol whose purpose is comparability: any N-TNT sample described according to it can be placed on the defect-state design map of Fig. 13 and compared with samples from other laboratories. The protocol is deliberately restricted to measurements that most materials laboratories already perform; its novelty lies in requiring that they be reported together and normalised consistently. Fig. 3 summarises the workflow and Table 3 lists the required items.

**Fig. 3:**
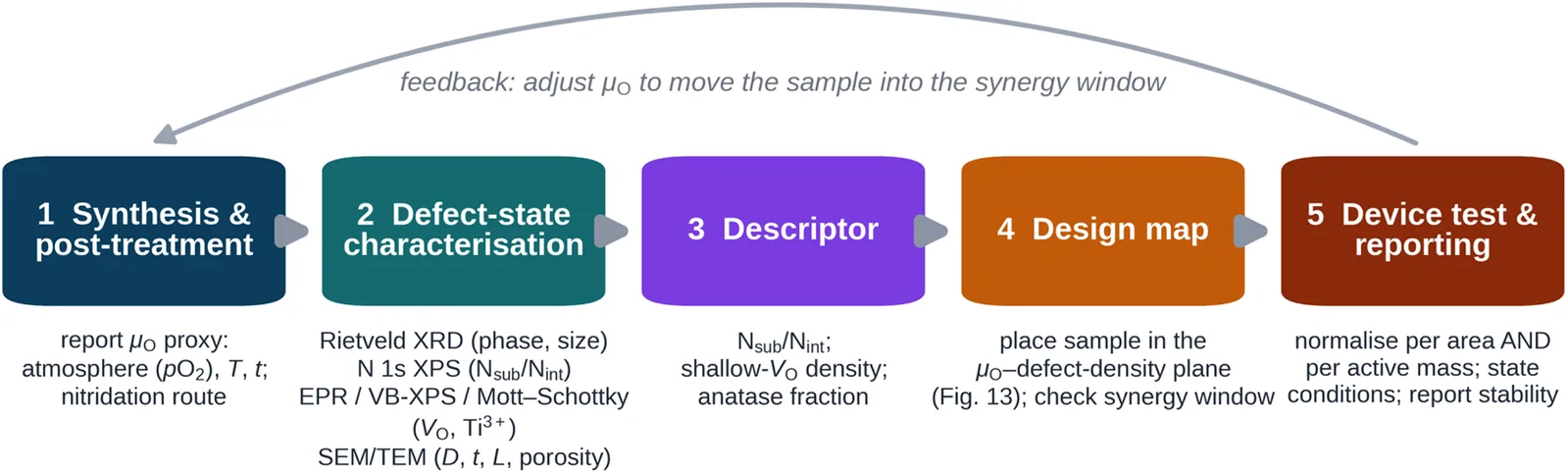
Workflow of the minimum defect-reporting protocol for N-TNT studies. Synthesis and post-treatment conditions are recorded as the *µ*_o_ proxy (step 1); the defect state is characterised by Rietveld XRD, N 1s XPS, EPR/VB-XPS/Mott–Schottky and SEM/TEM statistics (step 2); the defect-state descriptor is computed (step 3); the sample is located in the *µ*_o_-defect-density design map (step 4, Fig. 13); and device metrics are reported with dual normalisation and stability data (step 5). The feedback loop indicates adjustment of *µ*_o_ to move a sample into the synergy window.

**Table 3:** Minimum defect-reporting protocol for N-TNT studies: what to measure, by which method, what to report, and why

Quantity	Recommended method(s)	Minimum report	Why it matters
Phase composition, crystallite size	XRD with Rietveld refinement (state instrument, 2*θ* range, *R*-factors); Raman as complement	Anatase/rutile wt%; crystallite size (nm)	Phase sets band alignment, carrier lifetime and the anatase → rutile risk during nitridation
Nitrogen configuration	XPS N 1s deconvolution (state fitting model and constraints)	N_sub_ (≈396–397 eV) and N_int_ (≈399–400 eV) fractions; total N (at%)	N_sub_/N_int_ ratio decides band-edge states *vs.* mid-gap traps and couples to V_o_
Shallow V_o_/Ti^3+^ density	EPR (*g* ≈ 1.94–1.99; spin standard), VB-XPS/O 1s, Mott–Schottky	N_D_ (cm^−3^) or Ti^3+^ signal per mass; method stated	Governs conductivity, donor density and recombination; the core of the defect state
Nanotube geometry	SEM/TEM image statistics	*D*, *t*, *L*, porosity as mean ± SD (*n* ≥ 50); image-analysis method	Controls surface area, transport length and space-charge fraction
*µ*_o_ proxy	Process log	Atmosphere and pO_2_; *T*, ramp, dwell; nitridation route and dose (NH_3_ flow, plasma power/time, implantation fluence)	Fixes the defect state; needed to place the sample on the *µ*_o_ axis
Performance and stability	Application test (electrochemical, PEC, photocatalytic)	Normalised per geometric area and per active mass; illumination spectrum/intensity; electrolyte; potential window; retention after N cycles/h	Enables comparison across geometries, applications and laboratories

Three measurements define the defect state. Phase composition and crystallite size are obtained from Rietveld-refined XRD, reporting anatase and rutile weight fractions with refinement statistics rather than a qualitative ‘anatase-dominant’ label. The nitrogen configuration is obtained from deconvolved N 1s XPS spectra, reporting the fractions of substitutional (N_sub_, binding energy ≈396–397 eV) and interstitial (N_int_, ≈399–400 eV) nitrogen together with the total nitrogen content, so that the N_sub_/N_int_ ratio can be evaluated.^40^ The density of shallow oxygen vacancies and Ti^3+^ centres is obtained from at least one of EPR (Ti^3+^ signal at *g* ≈ 1.94–1.99, quantified against a spin standard), valence-band/O 1s XPS, or Mott–Schottky analysis (donor density N_D_), stating which method was used because the three probe different depths and different subsets of defects.^60,61^

Two further items make the measurements interpretable. The nanotube geometry—diameter *D*, wall thickness *t*, length *L* and porosity—must be reported as distributions (mean ± standard deviation, *n* ≥ 50) from SEM/TEM images, because *D*, *t* and *L* control surface area, transport length and the fraction of wall material within the space-charge region. And the *µ*_o_ proxy must be stated explicitly: the atmosphere and its oxygen partial pressure, the annealing temperature, heating rate and dwell time, and the nitridation route and dose (NH_3_ flow, plasma power and time, implantation fluence). Without these variables the defect state cannot be reproduced, and the sample cannot be located on the *µ*_o_ axis of the design map.

Finally, the evaluation strategy must allow performance to be compared across geometries and applications. Metrics should be normalised both per geometric electrode area and per active mass of TiO_2_; illumination spectrum and intensity, electrolyte composition and the tested potential window should be given; and stability should be reported as retention after a stated number of cycles or hours rather than as a qualitative statement. Adopting even a subset of these items would transform isolated record values into cumulative, comparable knowledge, and would allow the descriptor proposed in the conclusions—the triplet (N_sub_/N_int_, shallow-V_o_ density, anatase fraction)—to be computed for any published sample.

## 4. Synthesis methods for N-doped TiO_2_ nanotubes

The functional performance of N-TNT arrays is intrinsically governed by the synthesis pathway, which determines dopant configuration, defect density, phase composition, and morphological integrity. As summarized in Fig. 4, multiple fabrication strategies have been developed to engineer N incorporation into anodic TNTs, each providing distinct degrees of control over nitrogen chemical state, spatial distribution, and defect coupling.

**Fig. 4:**
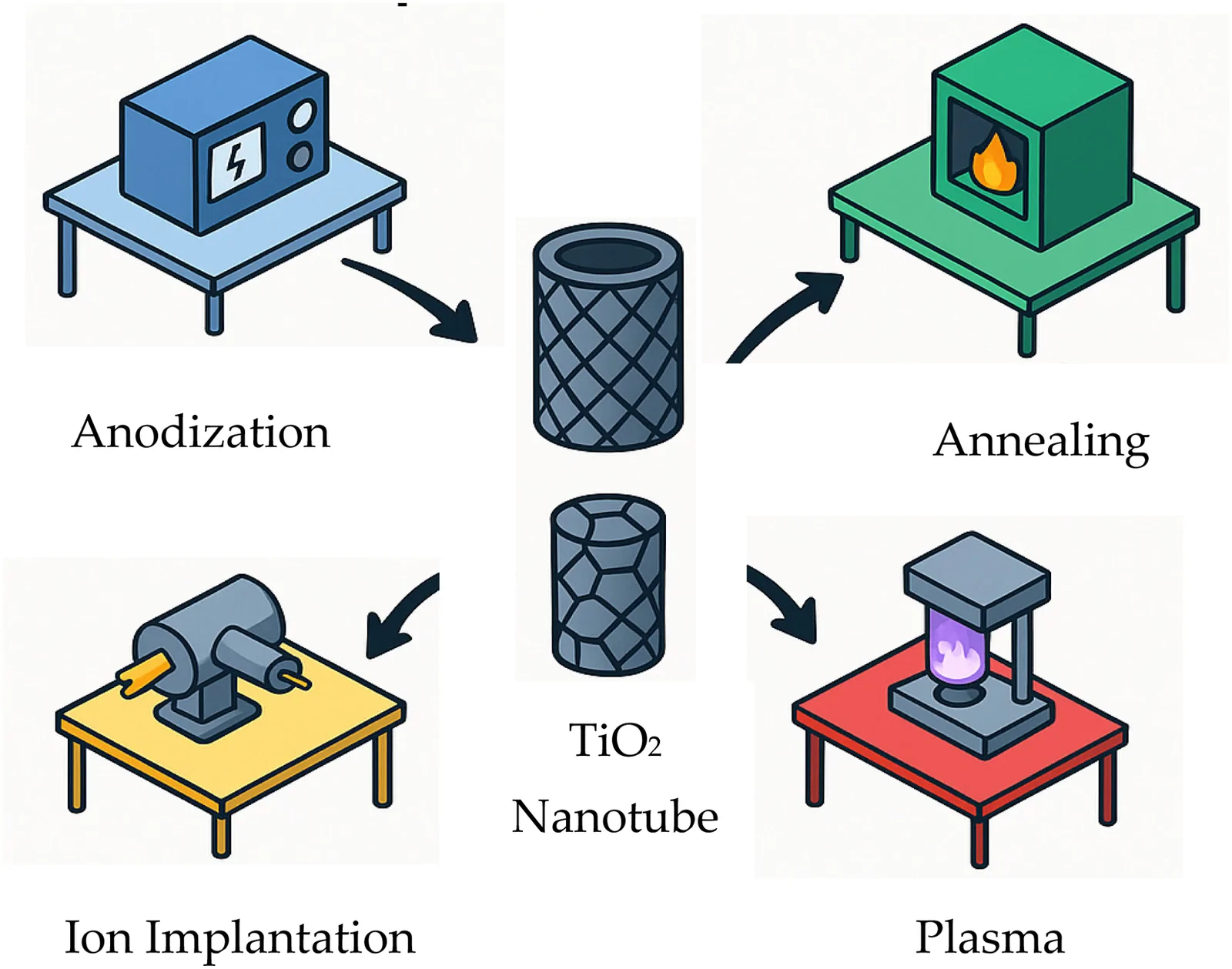
Synthesis methods for N-TNTs.

Among the most extensively investigated approaches are electrochemical anodization with subsequent nitridation, thermal annealing in nitrogen-containing atmospheres, ion implantation, and plasma-assisted treatments. These methods differ not only in processing conditions but also in their impact on substitutional *versus* interstitial nitrogen incorporation, oxygen-vacancy stabilization, and resulting photoelectrochemical behavior.

### 4.1 Nitrogen-doped TiO_2_ anodization

Electrochemical anodization remains the most established route for fabricating highly ordered TNT arrays directly on titanium substrates. In fluoride-containing electrolytes, nanotube formation proceeds through a dynamic competition between field-assisted oxide growth and localized chemical dissolution. Under anodic polarization, Ti is oxidized to Ti^4+^, which reacts with oxygen-containing species generated at the electrolyte-metal interface to form TiO_2_ (eqn (1)). Concurrently, fluoride ions promote complexation and dissolution of the oxide layer through the formation of soluble (TiF_6_)^2−^ species (eqn (2) and (3)), enabling self-organized tubular morphology development.^55^

1 Ti + 2H_2_O → TiO_2_ + 4H^+^ + 4e^−^

2 TiO_2_ + 4H^+^ + 6F^−^ → (TiF_6_)^2−^ + H_2_O

3 Ti^4+^ + 6F^−^ → (TiF_6_)^2−^

Nitrogen incorporation can be achieved either during anodization using nitrogen-containing electrolytes or through subsequent nitridation. For example, Li *et al.* synthesized N-TNT arrays in a glycerol/H_2_O/NH_4_F electrolyte followed by thermal annealing at 450 °C under controlled nitrogen flow (0–500 cc min^−1^), enabling tunable nitrogen contents up to 9.47 at%.^41^ The optimized N-TNAs exhibited a visible-light-driven methylene blue degradation rate constant of 0.26 h^−1^, representing a ∼125% enhancement over pristine TNAs (0.115 h^−1^), highlighting the strong sensitivity of photocatalytic kinetics to dopant concentration and post-treatment conditions.

Furthermore, two-step anodization protocols have been reported to improve nanotube ordering and diameter uniformity, thereby reducing structural heterogeneity and enhancing subsequent dopant distribution and photoelectrochemical response.^62^

### 4.2 Annealing method for N-doped TiO_2_ synthesis

Thermal annealing represents a controllable post-synthesis route for introducing nitrogen into pre-formed TNT architectures. When anodized TiO_2_ is treated in nitrogen-containing atmospheres (*e.g.*, N_2_, NH_3_, or Ar/N_2_ mixtures), nitrogen atoms diffuse into the lattice, occupying either substitutional sites (O → N replacement) or interstitial positions depending on temperature, gas composition, and treatment duration.^12,41^ Substitutional incorporation is typically associated with the formation of Ti–N bonds and the upward shift of the valence-band edge, resulting in bandgap narrowing and enhanced visible-light absorption. Simultaneously, annealing-induced defect chemistry promotes the formation of oxygen vacancies (V_o_) and Ti^3+^ centers, which can increase donor density and electrical conductivity when maintained within a controlled range.^14^ However, excessive defect generation or high-temperature treatment may introduce deep trap states and trigger partial anatase-to-rutile transformation, thereby compromising carrier lifetime and structural integrity.

For instance, Phuc Huo Lee *et al.* reported that annealing as-anodized TNT arrays at 450 °C for 3 h under N_2_ led to improved visible-light-driven methylene blue degradation (*λ* ≥ 400 nm, 120 mW cm^−2^) compared with pristine TNAs.^41^ The enhancement was attributed to a combined effect of band-edge modulation, defect-assisted charge separation, and improved carrier transport.

These findings underscore that annealing temperature and atmosphere must be carefully optimized to balance substitutional nitrogen incorporation with controlled vacancy formation, while avoiding phase instability and dopant redistribution.

### 4.3 Ion implantation for N-doped TiO_2_ synthesis

Ion implantation offers a high-precision post-synthesis strategy for introducing nitrogen into TNT arrays, enabling independent control over dopant dose, penetration depth, and spatial distribution. By tuning ion energy and fluence, nitrogen incorporation can be confined to the near-surface region-typically within several tens of nanometers-allowing selective modification of the electronically active interface.^63^ Depending on implantation parameters, nitrogen may occupy substitutional sites (forming Ti–N bonds) or interstitial positions associated with N–O linkages, each configuration producing distinct modifications in band structure and optical absorption behavior.^63,64^

In addition to dopant incorporation, energetic ion bombardment intrinsically generates oxygen vacancies (V_o_) and Ti^3+^ centers, which increase donor density and enhance charge separation when maintained within a controlled regime.^64,65^ However, excessive ion fluence can introduce lattice disorder, defect clustering, or partial amorphization, thereby reducing crystallinity and carrier mobility. For this reason, post-implantation annealing is commonly employed to recover structural order while preserving beneficial dopant-induced defect states.^63,65^

For example, Zhou *et al.* demonstrated that N-ion implantation into anodized TNTs, followed by mild annealing, significantly enhanced photoelectrochemical (PEC) activity under both UV and visible illumination. XPS analysis confirmed the coexistence of substitutional and interstitial nitrogen, with substitutional species prevailing at moderate implantation energies, highlighting the importance of energy-window optimization.^63^ More recent work has extended this approach to co-implantation strategies (*e.g.*, transition metal + N), enabling synergistic tuning of conductivity and light absorption.^65^

### 4.4 Plasma-assisted synthesis of nitrogen-doped TiO_2_

Plasma-assisted treatment provides a non-equilibrium route for nitrogen incorporation into TNT arrays, enabling controlled modification of surface chemistry and electronic structure at relatively low substrate temperatures. In nitrogen plasma environments generated by DC, RF, or microwave discharge systems, activated N species (N*, N^+^) interact with the TiO_2_ lattice, allowing incorporation in substitutional (Ti–N) or interstitial configurations depending on plasma power, exposure time, and pressure conditions.^55^

Unlike conventional thermal diffusion processes, plasma-assisted doping decouples nitrogen activation from bulk heating, thereby preserving nanotube morphology and minimizing unwanted anatase-rutile phase transformation. At the same time, the energetic plasma environment promotes the formation of oxygen vacancies (V_o_) and Ti^3+^ centers, which can enhance donor density and charge separation when maintained within a controlled regime.

Marcin Pesarek *et al.* demonstrated plasma-assisted nitridation of TNTs in a vacuum chamber directly coupled with X-ray photoelectron spectroscopy (XPS), enabling real-time monitoring of nitrogen chemical states. Their analysis confirmed the coexistence of substitutional and interstitial nitrogen species and highlighted the sensitivity of dopant configuration to plasma parameters.^40^ Compared with purely thermal or electrochemical approaches, plasma processing offers improved surface uniformity and defect-state control, making it particularly suitable for applications where interfacial charge-transfer kinetics dominate performance.

A comparative assessment of the principal doping routes-anodization-assisted nitridation, thermal annealing, ion implantation, and plasma treatment-is summarized in Table 4, providing a framework for selecting synthesis strategies based on target device requirements and defect-engineering objectives.

**Table 4:** Summary of doping strategies for TNTs, outlining the main advantages, limitations, applications, and resulting structures

No	Doping method	Advantages	Limitations	Best application	Resulting structure	Ref.
1	Anodization	Simple, low-cost, high geometric control	Mainly surface doping, requires post-annealing	Sensors, UV/visible photocatalysis	Well-aligned nanotube arrays with tunable length	21
2	Thermal annealing	Deep and uniform dopant diffusion, phase compatibility	Dopant inhomogeneity, phase distortion at high temperatures	Solar cells, energy storage devices	Stable anatase phase with embedded dopants	19
3	Ion implantation	Precise dose and depth control, tunable electronic properties	Requires advanced equipment, risk of lattice damage	PEC systems, wastewater treatment	Lattice-modified nanostructure with controlled defects	12
4	Plasma-assisted	Low-temperature process, good uniformity, minimal phase change	High cost, vacuum or RF systems needed	Visible-light photocatalysis, sensors	Uniform thin film with enhanced optical activity	40
5	Co-doping (*e.g.*, N + Metal)	Enhanced conductivity and optical response	Complex synthesis and dual-element control	LIBs, supercapacitors	Multi-phase network with high conductivity	66
6	C/N doping	Improved charge separation and electron transport	Structural inhomogeneity in some configurations	Photocatalysis, supercapacitors, DSSCs	TiO_2_ composites with graphene or carbon nitride	67

### 4.5 Comparative analysis of nitrogen-doping strategies for TiO_2_ nanotubes

The four principal nitrogen-doping approaches for TNT arrays-anodization-assisted incorporation, thermal annealing, ion implantation, and plasma treatment-differ fundamentally in dopant depth control, defect-state tunability, and structural impact. These differences directly influence electronic structure modulation and device-level performance.

Anodization-based routes provide geometric precision and scalability, enabling highly ordered nanotube arrays with optional *in situ* nitrogen incorporation *via* nitrogen-containing electrolytes. However, nitrogen introduced during anodization is often surface-biased and frequently interstitial in nature, limiting precise control over substitutional incorporation and oxygen-vacancy density without subsequent thermal treatment.^68^

Thermal annealing in nitrogen-rich atmospheres promotes deeper nitrogen diffusion and generally favors substitutional configurations (Ti–N bonding), leading to valence-band modulation and enhanced visible-light absorption. Controlled vacancy generation (Ti^3+^/V_o_) can improve donor density and carrier transport; nevertheless, excessive annealing may induce anatase-to-rutile transformation or dopant redistribution, adversely affecting phase stability and recombination dynamics.^41^

Ion implantation offers unparalleled control over dopant dose, depth profile, and associated defect chemistry. Depending on implantation parameters, both substitutional and interstitial nitrogen species can be introduced, accompanied by a controllable concentration of oxygen vacancies. The method is highly reproducible but requires specialized equipment, and high ion fluences may induce lattice disorder or partial amorphization, necessitating optimized post-implantation annealing to restore crystallinity without eliminating beneficial defects.^63^

Plasma-assisted nitridation provides a low-temperature, non-equilibrium alternative that preserves nanotube morphology while enabling surface-selective nitrogen incorporation. Plasma parameters allow fine tuning of surface defect density and dopant configuration; however, penetration depth is typically shallower than thermal diffusion processes, and system complexity may limit large-scale deployment.^69^

The trade-offs between these routes are summarised quantitatively in Table 5. Thermal annealing in NH_3_ or N_2_ delivers the deepest nitrogen incorporation—diffusion over the full wall thickness and, at sufficient time, along the tube length—and favours substitutional configurations, but only within a narrow temperature window: above roughly 550–600 °C the anatase → rutile transformation, grain coarsening and partial collapse of the tube walls set in, and the reducing atmosphere drives the material toward the over-reduced side of the design map with deep vacancies and metallic Ti^3+^.^27,41^ Plasma nitriding is its mirror image: the non-equilibrium, low-temperature process preserves the nanotube morphology and the as-crystallised phase, but nitrogen and the accompanying vacancies are confined to a layer of typically tens of nanometres, so that the bulk of the wall remains stoichiometric and the defect state is surface-localised.^69^ Ion implantation offers the most precise control of depth profile and dose but introduces lattice disorder that must be healed by a subsequent anneal, which partially re-equilibrates the defect population;^63^*in situ* incorporation during anodisation is the most scalable but yields predominantly interstitial nitrogen that is only converted to substitutional sites, and only coupled to V_o_, during the later thermal step.^68^

**Table 5:** Trade-off comparison of nitrogen-incorporation routes for TNT arrays, with the *µ*_o_ regime each route effectively imposes

Route	N penetration depth	Dominant N configuration	Morphology preserved	Anatase → rutile risk	Control over V_o_/Ti^3+^ (*µ*_o_ regime)	Typical conditions	Reproducibility/scalability	Ref.
*In situ* during anodisation	Shallow, surface-biased	Mostly N_int_	Yes (native)	Low (as-formed amorphous; set by later anneal)	Weak—*µ*_o_ fixed by subsequent crystallisation	N-containing electrolyte, room temperature	High/high	68
Thermal annealing (NH_3_/N_2_)	Deep—full wall thickness, µm along tube	N_sub_-dominant at moderate *T*	Yes up to ≈500–550 °C; coarsening/collapse above	High above ≈600 °C	Strong—uniformly low *µ*_o_; risk of over-reduction	400–600 °C, 1–4 h, NH_3_ or N_2_ flow	High/high	27 and 41
Ion implantation	Tunable profile, tens-hundreds of nm	N_sub_ + N_int_, dose-dependent	Yes, but lattice damage/amorphisation at high fluence	Low; post-anneal required	Moderate—implantation-induced V_o_ relax during anneal	10–100 keV, 10^15^–10^17^ ions cm^−2^	High/low (equipment)	63
Plasma nitriding	Shallow, tens of nm	Surface N_sub_/N_int_ mixture	Yes (low temperature)	Low	Surface-localised low *µ*_o_; bulk stoichiometric	RF/DC plasma, N_2_ or NH_3_, RT-300 °C, minutes	Medium/medium	69

Viewed through the framework of this review, the four routes differ primarily in the *µ*_o_ they impose and in the depth over which they impose it. Thermal nitridation sets a low, uniform *µ*_o_ throughout the material; plasma treatment sets a low *µ*_o_ in a thin skin; implantation sets a non-equilibrium defect population that relaxes toward the *µ*_o_ of the healing anneal; and anodisation leaves *µ*_o_ to be fixed entirely by the subsequent crystallisation step. Method selection is therefore not a question of which route is ‘best’ but of which depth profile of the defect state the application requires—bulk for lithium storage and photoelectrochemistry, surface for photocatalysis and sensing—and of the processing window within which that profile can be reached without leaving the synergy window.

From a design perspective, method selection should be application-driven. Surface-dominated processes such as sensing or interfacial photocatalysis may benefit from plasma-assisted or anodization-mediated incorporation, whereas bulk-sensitive applications-including photoelectrochemical water splitting and lithium-ion storage-often require deeper dopant penetration and controlled vacancy engineering achievable *via* thermal annealing or ion implantation.

Ultimately, rational tuning of dopant configuration, defect density, and phase composition-rather than the synthesis route alone-governs the functional performance of N-TNTs. The following section evaluates how these engineered architectures translate into measurable performance across energy and environmental systems.

### 4.6 Formation and regulation of defects in N-doped TiO_2_ nanotubes

Nitrogen incorporation in TNT arrays extends beyond bandgap modulation and functions as a defect-engineering mechanism that governs oxygen-vacancy (V_o_) density, Ti^3+^ stabilization, and electronic state redistribution. Both experimental and computational studies demonstrate that nitrogen dopants reduce the formation energy of oxygen vacancies, thereby promoting their coexistence within the lattice and shifting the density of states toward the Fermi level.^70^ This coupling modifies carrier concentration, band bending, and visible-light absorption.

A defect “synergy window” emerges as a function of nitrogen concentration (Fig. 5). In pristine TiO_2_, intrinsic V_o_ and Ti^3+^ populations are minimal. Moderate nitrogen incorporation promotes controlled V_o_/Ti^3+^ generation, increasing donor density and enhancing charge separation efficiency. In contrast, excessive nitrogen doping results in defect overpopulation, generating deep mid-gap trap states that accelerate recombination and degrade PEC performance.

**Fig. 5:**
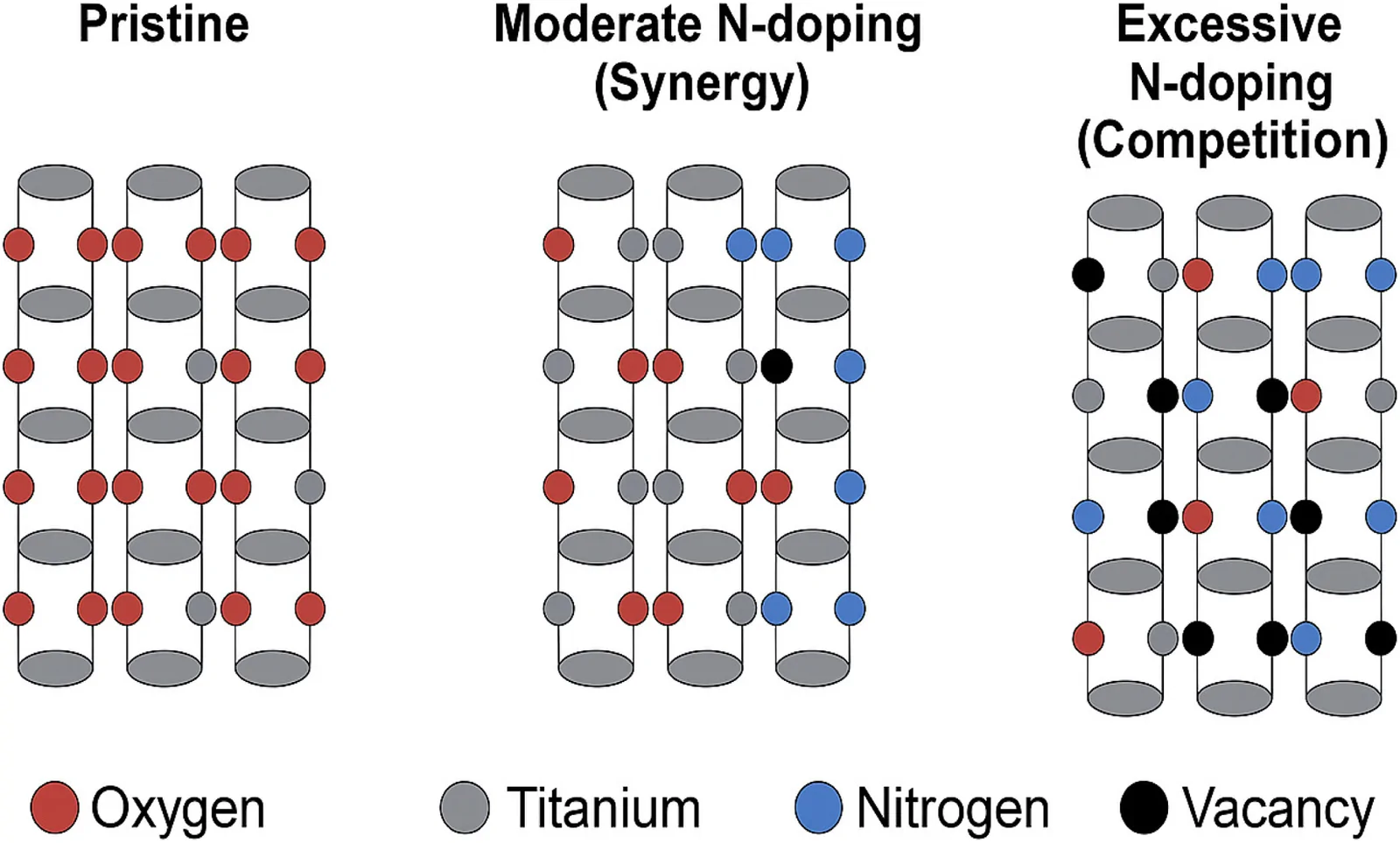
Effect of nitrogen doping level on defect formation in TNTs: pristine, moderate (synergy), and excessive (competition).

First-principles DFT calculations consistently show that substitutional nitrogen (N_o_) introduces N 2p states near the valence band maximum, narrowing the bandgap and enabling visible-light activation.^71^

Substitutional N also lowers the formation energy of V_o_ by up to ∼0.5 eV, stabilizing Ti^3+^ centers and increasing carrier density.^72^ Projected density-of-states (PDOS) and spin-density analyses reveal that substitutional N produces partially delocalized states hybridized with O 2p orbitals, whereas interstitial N (N_i_) introduces localized mid-gap states prone to recombination.^43,71,73^

The calculated spin-density maps reveal that substitutional nitrogen produces more delocalized electronic states, whereas interstitial nitrogen generates localized defect levels within the band gap, which can act as recombination centers (Fig. 6).

**Fig. 6:**
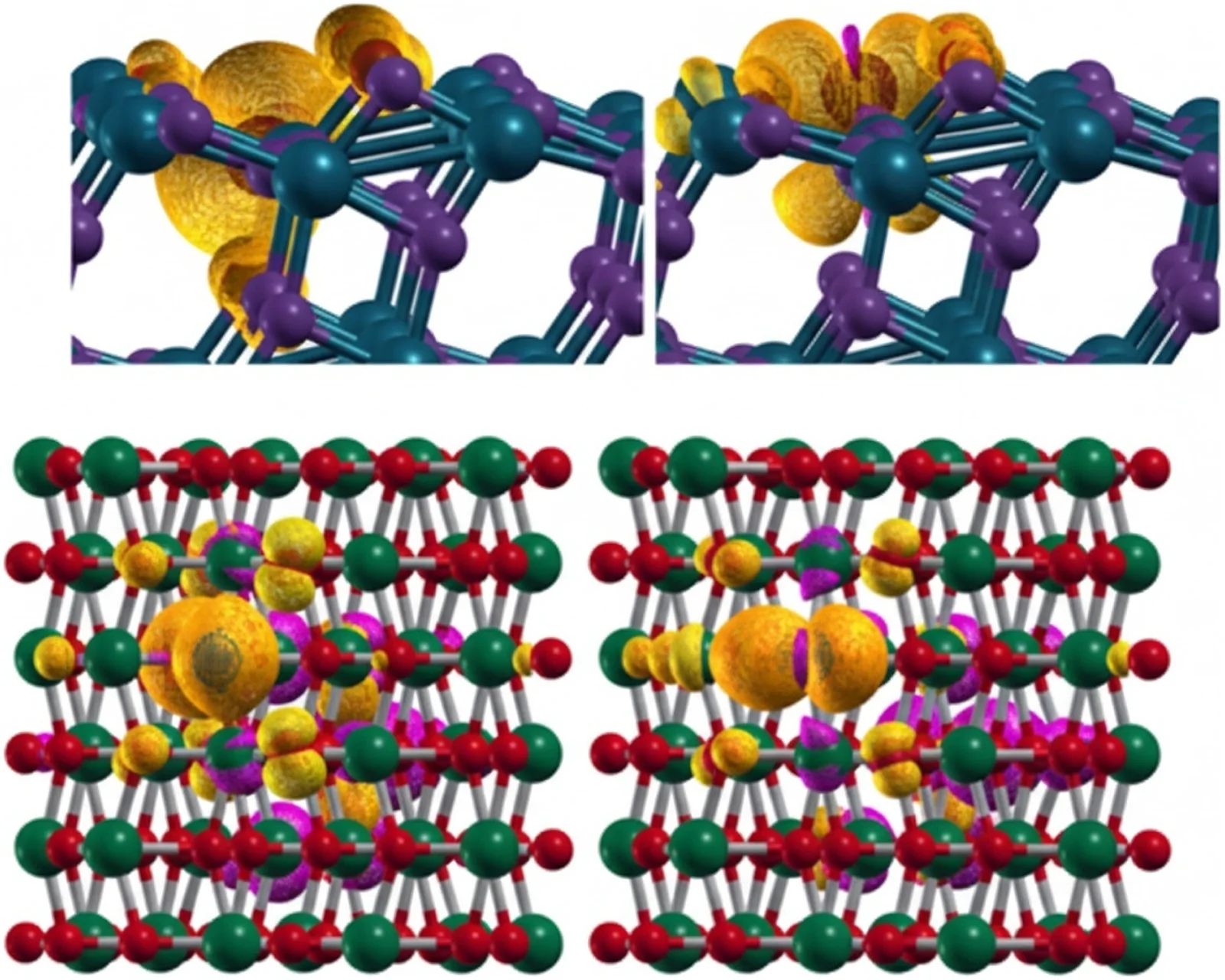
Spin-density distributions of TiO_2_ containing substitutional (N_o_) and interstitial (N_i_) nitrogen dopants, illustrating the formation of delocalized electronic states for N_o_ and localized mid-gap states for N_i_. Adapted from Fig. 12 of ref. 73 (S. A. Kakil, H. Y. Abdullah, T. G. Abdullah and N. Manini, Subsurface depth dependence of nitrogen doping in TiO_2_ anatase: a DFT study, *Journal of Physics: Condensed Matter*, 2021, **33**(20), 205703, DOI: https://doi.org/10.1088/1361-648X/abce41) with permission from IOP Publishing, copyright 2021. © IOP Publishing. Reproduced with permission. All rights reserved.

Surface-focused studies further confirm reduced V_o_ formation energy and modified surface electronic states upon substitutional N incorporation.^74^

Experimental spectroscopy corroborates these computational predictions. XPS consistently identifies substitutional N *via* N 1s components near ∼396 eV, often accompanied by Ti^3+^ shoulders in Ti 2p spectra, confirming vacancy stabilization.^75^ Progressive nitrogen incorporation produces bandgap narrowing, red-shifted absorption edges, Raman *E*_g_-mode broadening, and increased defect density (Fig. 7).^67,72^

**Fig. 7:**
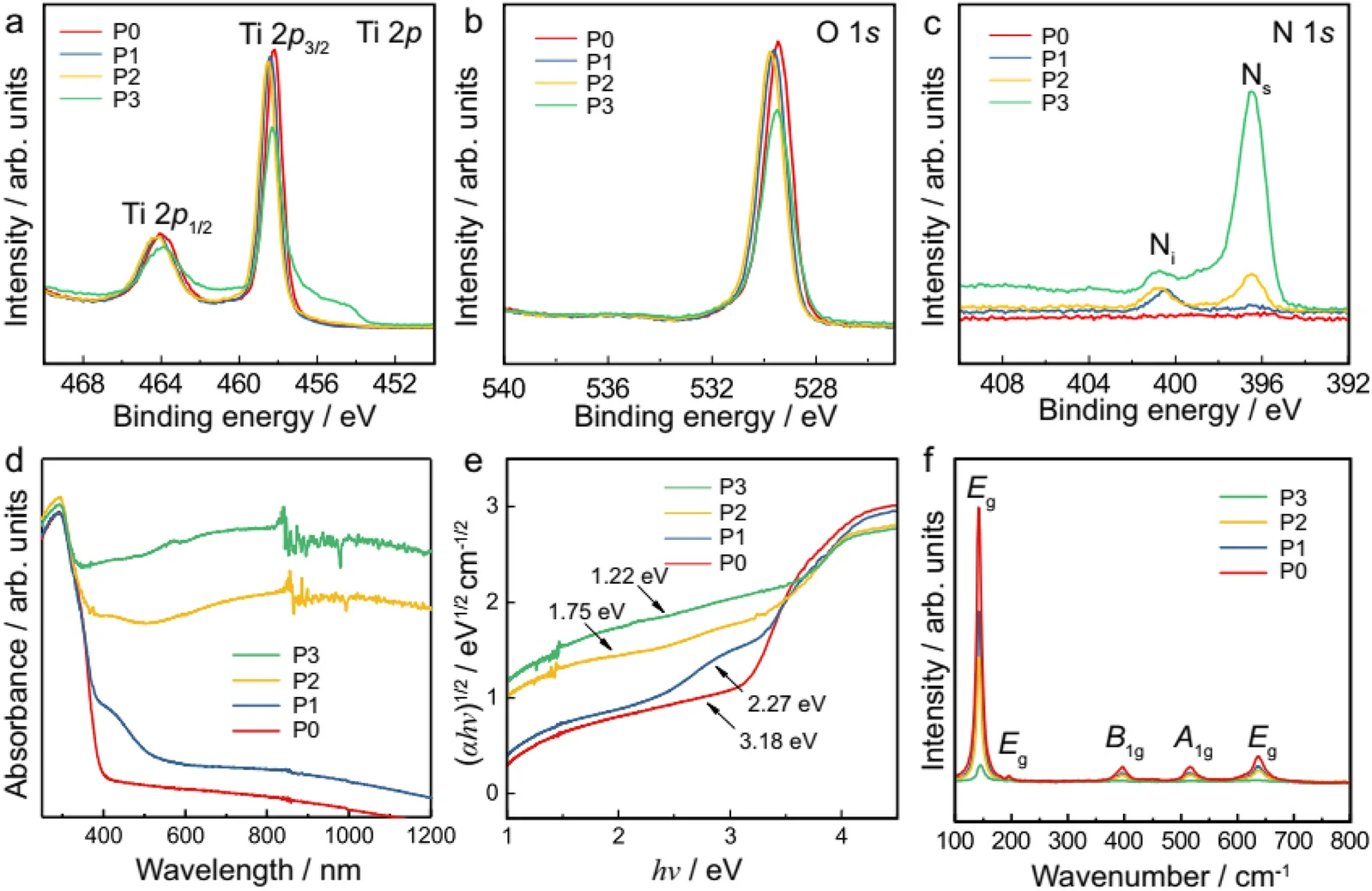
XPS, UV-vis, and Raman characterization of N-doped TiO_2_ samples (P0–P3): (a) Ti 2p, (b) O 1s and (c) N 1s XPS spectra, (d) UV-vis absorbance spectra, (e) Tauc plots and (f) Raman spectra, showing Ti^3+^ formation, substitutional/interstitial N states, bandgap narrowing, and defect-induced crystallinity changes.^72^ Reproduced from ref. 72 (Nature Communications, Springer Nature) under the terms of the Creative Commons Attribution 4.0 licence (CC BY 4.0).

Electron paramagnetic resonance (EPR) directly detects N-related radical species coupled to Ti^3+^ centers, linking defect formation to enhanced visible-light absorption and charge lifetime extension.^75,76^

Photoluminescence (PL/TRPL) measurements further show reduced recombination intensity at moderate doping levels, while excessive doping reintroduces trap-assisted decay pathways.^72,75,76^

Collectively, computational and experimental evidence converge on a central principle:✓ Substitutional nitrogen (N_o_) + shallow V_o_/Ti^3+^ → beneficial defect regime✓ Excess interstitial nitrogen (N_i_) + deep traps → recombination regime

Optimized N-TNTs therefore require controlled substitutional incorporation and moderated vacancy density, ensuring bandgap narrowing without trap proliferation. Maintaining this defect balance is critical for sustained PEC activity and long-term structural stability.

### 4.7 Computational insights into nitrogen incorporation and defect energetics in TiO_2_ nanotubes

Density functional theory has shaped the understanding of nitrogen-doped TiO_2_ since the original proposal that substitutional nitrogen narrows the band gap by mixing N 2p with O 2p states at the top of the valence band.^12^ Subsequent work, combining DFT with EPR, established a more nuanced picture in which substitutional (N_sub_) and interstitial (N_int_) nitrogen give rise to distinct localised states—N_sub_ producing states just above the valence-band maximum and N_int_ producing higher-lying π*-type states of the N–O species—and in which the paramagnetic nitrogen centres detected under visible light are the photoactive species.^15,31^ The photoactivity is polymorph-dependent: the same nitrogen states lie closer to the valence band in anatase than in rutile, so that gap narrowing in anatase becomes gap contraction from below in rutile.^33^Table 6 summarises the computational studies most relevant to N-TNT engineering.

**Table 6:** Representative computational (DFT) studies underpinning the defect-engineering framework for N-doped TiO_2_ and TiO_2_ nanotubes

Study (ref.)	System/model	Method	Key finding relevant to N-TNT engineering
Asahi *et al.*, 2001 (ref. 12)	Substitutional N in anatase (bulk)	FLAPW (LDA)	N 2p states mix with O 2p at the valence-band top → visible-light absorption; substitutional N most effective
Livraghi *et al.*, 2006 (ref. 15)	N-doped TiO_2_	DFT + EPR under visible light	Paramagnetic N-centred states are the visible-light photoactive centres; their population couples to V_o_
Di Valentin *et al.*, 2007 (ref. 31)	N_sub_, N_int_ and N–V_o_ complexes in anatase	DFT (GGA/hybrid) + EPR	N_sub_ and N_int_ give distinct localised states; N lowers the V_o_ formation energy—theoretical basis of the stabiliser role of nitrogen
Di Valentin, Pacchioni & Selloni, 2004 (ref. 33)	N_sub_ in anatase *vs.* rutile	DFT	Polymorph-dependent position of N states → different photoactivity of N-doped anatase and rutile
Na-Phattalung *et al.*, 2006 (ref. 35)	Native defects in anatase	DFT, formation energies *vs. µ*_o_	V_o_ and Ti interstitials dominate as donors under O-poor conditions; formation energies fall with decreasing *µ*_o_
Morgan & Watson, 2007 (ref. 77)	V_o_ at rutile (110)	DFT + *U*	Excess electrons localise as Ti^3+^ with gap states below the CB in DFT + *U*, but delocalise in GGA—shallow/deep character depends on correlation treatment
Finazzi *et al.*, 2008 (ref. 60)	Reduced bulk anatase	GGA/GGA + *U*/hybrid	Same conclusion for anatase: localisation of Ti^3+^ states is functional-dependent; defines the shallow-donor regime relevant to conductivity
Di Valentin, Pacchioni & Selloni, 2009 (ref. 61)	Reduced and n-doped TiO_2_ (V_o_, Ti_int_, H, Nb)	Hybrid DFT	Ti^3+^ species have a common signature regardless of donor identity → Ti^3+^ density is the appropriate observable
Bandura & Evarestov, 2009 (ref. 78)	Anatase (101)-derived nanotubes	First-principles LCAO	Curvature- and diameter-dependent strain energy and band gap of TiO_2_ nanotubes → geometry modulates defect energetics

The result of greatest consequence for this review is the coupling between nitrogen and oxygen vacancies. Calculations show that substitutional nitrogen substantially lowers the formation energy of an adjacent oxygen vacancy, so that N-doped TiO_2_ equilibrates with a higher V_o_ population than undoped TiO_2_ under the same oxygen chemical potential;^31^ conversely, the excess electrons released by V_o_ can compensate the nitrogen acceptor, coupling the two defects electronically as well as thermodynamically. This is the theoretical basis for the stabiliser role of nitrogen: nitrogen does not create the functional defect state by itself, but shifts the *µ*_o_ window within which a given V_o_/Ti^3+^ population is stable.

Formation-energy diagrams of native defects provide the second pillar. Within the standard supercell formalism, the formation energy of an oxygen vacancy decreases linearly with decreasing oxygen chemical potential, and V_o_ and titanium interstitials are the dominant donors in anatase under oxygen-poor conditions.^35^ Because *µ*_o_ enters these diagrams explicitly, they are the natural theoretical counterpart of the design map of Fig. 13: moving along the *µ*_o_ axis of the map corresponds to moving along the horizontal axis of a defect-formation-energy diagram. The character of the resulting Ti^3+^ states—shallow, delocalised donors or deep, localised polarons—is sensitive to the treatment of electron correlation: standard GGA delocalises the excess electrons, whereas DFT + *U* and hybrid functionals localise them on Ti^3+^ sites and place the corresponding states in the gap.^60,77^ The nature of the Ti^3+^ species is nevertheless largely common to different donors (V_o_, Ti interstitials, hydrogen, niobium), which is why a single Ti^3+^ signature, rather than the identity of the donor, is the appropriate experimental observable.^61^

Calculations on explicit nanotube models add the geometric dimension. Nanotubes constructed by rolling anatase (101) layers show curvature- and diameter-dependent strain energies and band gaps, with the gap approaching the bulk value for large diameters and the thinnest walls behaving as quasi-two-dimensional layers.^78^ These results imply that the wall thickness *t* and diameter *D* of an anodic nanotube modulate not only the surface area and transport length but also the defect formation energies and band-edge positions of the wall material—which is why geometry appears as the second axis of the design map alongside *µ*_o_.

Taken together, the computational literature supports each element of the framework proposed here: the *µ*_o_ dependence of the V_o_/Ti^3+^ population, its stabilisation by substitutional nitrogen, the distinction between shallow and deep Ti^3+^ states, and the geometric modulation introduced by the nanotube wall. What is still missing is a calculation that treats nitrogen, oxygen vacancies and nanotube curvature simultaneously as a function of *µ*_o_; such a study would directly test the position and width of the synergy window predicted in this review.

## 5. Applications of doped TiO_2_ nanotubes

The functional relevance of N-TNT arrays arises from the coordinated modulation of band structure, defect chemistry, and one-dimensional charge-transport pathways established in Sections 3 and 4. Nitrogen incorporation narrows the effective bandgap through N 2p-O 2p hybridization while simultaneously stabilizing controlled Ti^3+^/V_o_ populations that enhance donor density and carrier separation. Coupled with the vertically aligned nanotubular morphology-which provides high surface area, directional electron transport, and improved electrolyte accessibility-these effects translate into measurable performance gains across energy and environmental systems.

Consequently, N-TNTs have been investigated in application domains where light absorption, interfacial charge transfer, and defect-mediated transport govern device efficiency. These include photocatalysis and environmental remediation, PEC water splitting, lithium-ion and sodium-ion storage, supercapacitors, and related solar energy conversion platforms.

The following subsections examine these applications from a structure–property–performance perspective, emphasizing how nitrogen configuration, defect density, and nanotube geometry collectively determine functional output. A schematic overview of these application domains is presented in Fig. 8.

**Fig. 8:**
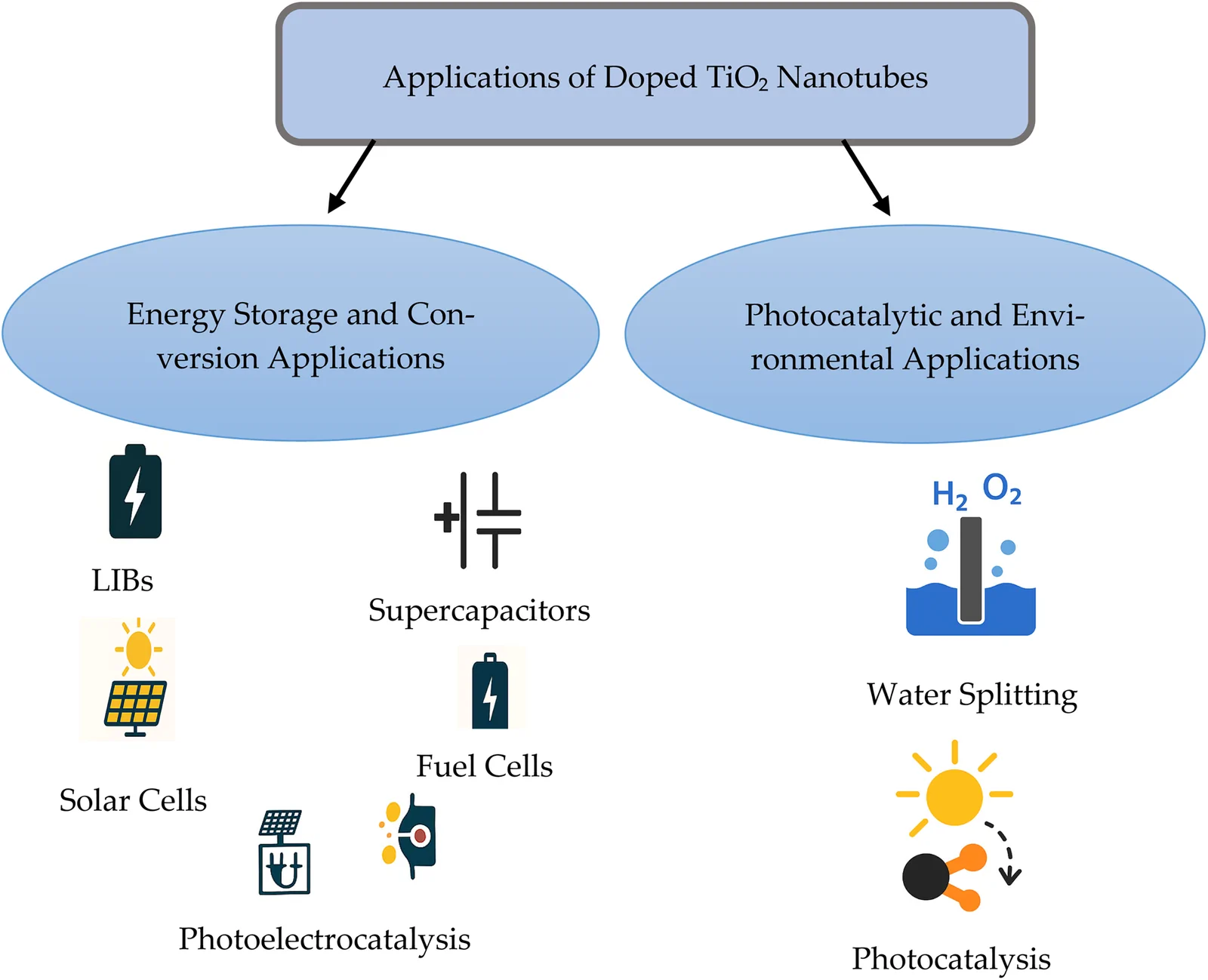
Applications of doped TNTs.

### 5.1 Energy storage and conversion applications

#### 5.1.1 Lithium-ion batteries (LIBs)

Lithium-ion batteries (LIBs) remain the dominant electrochemical energy storage technology due to their high energy density, operational efficiency, and scalability across portable electronics, electric vehicles, and renewable energy systems. While graphite is the commercial standard anode, concerns related to safety, lithium plating, and long-term stability have motivated the exploration of alternative materials. TiO_2_-based anodes have emerged as intrinsically safer candidates owing to their higher lithiation potential (∼1.5–1.8 V *vs.* Li/Li^+^), structural robustness, low toxicity, and cost-effectiveness.^79^ Unlike lithium titanate (Li_4_Ti_5_O_12_), which operates *via* zero-strain intercalation, TiO_2_ polymorphs rely on reversible Li^+^ insertion within anatase/rutile frameworks and are fundamentally distinct in structure and electrochemical behavior. In particular, ordered TNT architectures provide shortened Li^+^ diffusion paths, improved electrolyte accessibility, and enhanced mechanical stability during cycling.^80^

Despite these advantages, pristine TiO_2_ suffers from limited electronic conductivity and moderate Li^+^ diffusivity, restricting high-rate performance. Nitrogen doping and nanotube geometry engineering therefore serve as complementary strategies to enhance carrier density, reduce charge-transfer resistance, and improve reversible capacity, forming the basis for the advanced anode systems discussed in the following sections.

Anatase-phase TiO_2_ has been widely investigated as a lithium-ion battery anode owing to its structural stability and relatively high lithiation potential. However, its practical capacity is constrained by moderate Li^+^ diffusivity, intrinsically low electronic conductivity, and structural distortion associated with the tetragonal-to-orthorhombic transition during lithiation.^81^

To address these limitations, nanoscale engineering has been employed to shorten diffusion pathways and enhance interfacial kinetics. One-dimensional architectures such as nanotubes and nanorods provide increased surface area, reduced Li^+^ transport distances, and lower charge-transfer resistance compared to bulk TiO_2_.^82^

Beyond geometric optimization, heteroatom doping-particularly nitrogen incorporation-has emerged as a defect-engineering strategy to enhance conductivity and interfacial charge transfer. Substitutional nitrogen introduces donor states and stabilises Ti^3+^/V_o_ populations, thereby reducing *R*_ct_ and facilitating lithium-ion transport when maintained within a controlled concentration range.^83^

Hybridization with conductive scaffolds further amplifies these effects. For instance, nitrogen-doped TiO_2_/rGO composites demonstrated 318 mAh g^−1^ initial capacity with retention of 210 mAh g^−1^ after 2000 cycles, reflecting improved electronic percolation and interfacial transport.^84^

Similarly, N-doped TiO_2_-B/graphene systems exhibited high initial capacities (583.3 mAh g^−1^) and strong rate capability (153.3 mAh g^−1^ at 50C), attributed to synergistic structural stabilization and enhanced carrier mobility.^85^

*In situ* TEM studies provide mechanistic confirmation that nitrogen incorporation helps preserve crystallographic coherence during multi-stage lithiation, mitigating structural degradation and supporting long-term cycling stability.^29^

Collectively, these findings indicate that the performance of N-doped TiO_2_ anodes arises from coordinated optimization of nanotube geometry, substitutional nitrogen incorporation, controlled defect density, and conductive network integration.

Across the studies summarized in Table 7, several consistent structure–property–performance trends can be identified for high-performance N-TNT anodes.^19,20,29,86^ Architectures featuring moderately long nanotubes with well-defined and open channels generally deliver higher reversible capacities at practical C-rates compared to either very short or excessively long tubes. This reflects a critical balance between accessible surface area and minimized electron/ion transport distances.^20,29^ In addition, systems in which nitrogen is incorporated predominantly in substitutional form-accompanied by a controlled population of Ti^3+^/oxygen-vacancy (V_o_) defects and an anatase-rich or mixed anatase/rutile phase-consistently exhibit reduced charge-transfer resistance (*R*_ct_) and improved rate capability relative to undoped TNTs.^19,86^ Integration with conductive backbones such as reduced graphene oxide (rGO) or carbon nanofibers further enhances electronic percolation pathways, enabling stable cycling at elevated current densities and mitigating polarization losses.^86,87^ Collectively, these findings demonstrate that coordinated optimization of nanotube geometry, phase composition, and nitrogen-induced defect chemistry is more effective than isolated parameter tuning when designing high-performance N-doped TiO_2_ anodes for lithium-ion batteries.^19,20,29,86,87^

**Table 7:** Engineering-oriented structure-defect-performance matrix of N-TNT anodes for LIBs

System/architecture	Dominant N configuration	Defect engineering (Ti^3+^/V_o_)	Conductive enhancement	Specific capacity	Rate capability	Cycling stability	Ref.	Stage (M/O/A)
N-TiO_2_/rGO hybrid	Substitutional N + N–C coupling	Moderate Ti^3+^, shallow V_o_	rGO electron percolation	318 → 210 mAh g^−1^ (2000 cycles)	Stable at practical C-rates	∼66% retention	84	O
Few-layer TiO_2_-B/N-graphene	Substitutional-dominant	Controlled defect density	Graphene backbone	583.3 → 326.4 mAh g^−1^ (100 cycles)	153.3 mAh g^−1^ @ 50C	∼67.8% retention	85	O
N-doped anatase TNTs	Substitutional N (NH_3_ treated)	Reduced *R*_ct_, V_o_-assisted transport	Self-supported TNT array	∼200 @0.1C; ∼100 @10C	High-rate stability	Stable cycling	29	O
N-TNTs (areal)	NH_3_-cracked N incorporation	Ti^3+^-assisted Li^+^ diffusion	Direct growth on Ti substrate	975 µAh cm^−2^ initial	Excellent rate performance	>98% coulombic efficiency	19	O
Thermally N-TNTs	Ti–N bonding (controlled anneal)	Moderate vacancy tuning	—	Enhanced *vs.* pristine TNT	Improved rate retention	Improved cycling	30	O
TiO_2_/N-doped carbon-Si hybrid	N-doped carbon interface	Interface-stabilized defects	Carbon conductive matrix	Enhanced *vs.* pristine Si	Stable under high load	>200 cycles stable	88	O
TiO_2_/N-doped CNF composite	Substitutional N + CNF support	Improved electronic pathways	Conductive CNF matrix	High capacity (low rate)	Improved kinetics	Good retention	89	O
Mn/F/N co-doped TiO_2_(B)	Multi-element substitution	Synergistic defect tuning	Intrinsic improvement	Stable under high current	>200 cycles retention	Good stability	90 and 91	O

##### 5.1.1.1. Critical assessment by research stage

The lithium-storage literature on N-TNTs is dominated by performance-optimisation studies (stage O in Table 7) that report capacity and cycling metrics for a particular synthesis, whereas mechanism-oriented work (stage M) that quantifies the shallow-V_o_/Ti^3+^ density and relates it to the measured rate capability is scarce, and practical-application studies (stage A) on full cells, mass loading and calendar ageing are almost absent. Two contradictions follow. Improvements are attributed interchangeably to ‘enhanced conductivity from nitrogen’ and to ‘oxygen vacancies’, without measurements that could separate the two; and the same nitrogen loading is reported as beneficial in one study and detrimental to rate capability in another, which is expected if the samples occupy different positions on the *µ*_o_ axis but is untestable from the data as reported. Until donor density and post-treatment atmosphere are reported alongside capacity, the field cannot distinguish a genuine nitrogen effect from a *µ*_o_ effect.

#### 5.1.2 Supercapacitors

N-TNTs have emerged as promising electrode materials for electrochemical supercapacitors owing to their synergistic combination of one-dimensional architecture, tunable defect chemistry, and structural stability. The incorporation of nitrogen into the TiO_2_ lattice modifies the electronic band structure, increases carrier density, and promotes the formation of oxygen vacancies (V_o_) and Ti^3+^ centers. These changes collectively enhance electrical conductivity and redox activity, enabling improved charge storage through both electric double-layer capacitance (EDLC) and pseudocapacitive mechanisms.^18,19^

Compared to pristine TNTs, N-TNTs generally exhibit higher specific capacitance, improved rate capability, and superior cycling stability. Raj and Prasanth (2018) reviewed TNT-based supercapacitor electrodes and reported specific capacitances approaching 150 F g^−1^ depending on morphology and electrolyte conditions.^92^ Building upon this baseline, Appadurai *et al.* (2019) demonstrated that N-TNTs prepared *via* anodization exhibited enhanced double-layer capacitance and reduced charge-transfer resistance, achieving ∼835 µF cm^−2^ (∼417 F g^−1^).^19^ These improvements were attributed to increased electronic conductivity and defect-assisted surface charge accumulation.

A more aggressive defect-engineering strategy was reported by Ghorbani and Khameneh Asl (2021), who combined nitrogen doping with electrochemical reduction, producing nanotube electrodes with capacitance values exceeding ∼5666 µF cm^−2^ (∼2833 F g^−1^).^18^ The substantial enhancement was associated with controlled oxygen-vacancy generation and improved electron transport pathways, which amplified pseudocapacitive contributions while maintaining structural integrity. These findings highlight the importance of balancing dopant configuration and defect density to avoid excessive recombination or structural degradation.

Hybridization strategies further extend performance. Yang *et al.* (2023) integrated N-doped TiO_2_ with Ti_3_C_2_T_*x*_ MXene and graphene into a three-dimensional interconnected architecture, achieving high capacitance and stability in ionic-liquid electrolytes.^93^ Similarly, Akbar *et al.* (2024) fabricated ZnSe-TiO_2_ composites embedded with nitrogen-doped graphene nanosheets, delivering 222C g^−1^ with excellent cycling robustness.^94^ In these systems, nitrogen doping enhances electrical conductivity and introduces additional redox-active sites, while conductive carbon or MXene frameworks provide efficient electron percolation networks.

Table 8 summarizes representative studies on N-TNT-based supercapacitor electrodes, highlighting correlations between synthesis route, defect chemistry, and electrochemical performance.^18,19,82–84^ A recurring trend is that moderate substitutional nitrogen incorporation-accompanied by controlled oxygen-vacancy populations-consistently improves capacitance and rate performance. In contrast, excessive defect densities may increase internal resistance or compromise long-term stability. Furthermore, integrating N-TNTs with conductive scaffolds (*e.g.*, graphene, carbon nanofibers, or MXene) significantly enhances electron transport and mechanical resilience under high current densities.

**Table 8:** Summary of studies on N-TNTs as supercapacitor electrodes

Architecture/system	Charge storage mechanism	Dominant N/defect role	Specific capacitance	Rate/stability behavior	Ref.	Stage (M/O/A)
TNT electrodes (reviewed systems)	Primarily EDLC + limited pseudocapacitance	Intrinsic surface states	Up to ∼150 F g^−1^	Good cycling stability	28	M
N-TNTs (anodized)	EDLC + defect-assisted pseudocapacitance	Substitutional N + moderate V_o_/Ti^3+^	∼835 µF cm^−2^ (∼417 F g^−1^)	Improved conductivity and stable cycling	19	O
N-TNTs (N-doping + electrochemical reduction)	Enhanced pseudocapacitance	High V_o_ density + Ti^3+^ enrichment	∼5666 µF cm^−2^ (∼2833 F g^−1^)	Strong rate performance, high retention		O
Ti_3_C_2_T_*x*_/TiO_2_-graphene hybrid	Surface redox + conductive synergy	N-modified interfaces + defect coupling	High performance (ionic liquid)	Improved structural stability	18	O
ZnSe-TiO_2_ with N-doped graphene	Hybrid EDLC + redox pseudocapacitance	N-enhanced conductivity + active sites	222 C g^−1^	Robust cycling stability	92	O

In summary, nitrogen doping serves as a controllable defect-engineering strategy that enhances conductivity, redox activity, and interfacial charge transport in TNT electrodes. When optimized in conjunction with nanotube geometry and hybrid architectures, N-TNTs represent a viable platform for next-generation supercapacitor systems with improved energy-power balance and cycling durability.^19,95^

##### 5.1.2.1. Critical assessment by research stage

Supercapacitor studies (Table 8) are almost exclusively stage O, and the very large spread of reported capacitances—from ≈150 F g^−1^ for reviewed TNT systems to values an order of magnitude higher after nitrogen doping combined with electrochemical reduction—reflects differences in the defect state and in normalisation practice as much as in intrinsic material quality. The mechanistic interpretation is frequently circular: pseudocapacitance is attributed to Ti^3+^/V_o_, and Ti^3+^/V_o_ are inferred from the pseudocapacitance. Stage-M work that measures N_D_ independently (by Mott–Schottky or EPR) and stage-A work on self-discharge, leakage and long-term float stability are both needed; without them the impressive areal capacitances cannot be translated into device claims.

#### 5.1.3 Solar cells (including DSSCs)

Dye-sensitized solar cells (DSSCs) have attracted sustained attention as low-cost photovoltaic systems, in which the semiconductor photoanode plays a decisive role in light harvesting and electron transport. Among various architectures, TNT arrays offer a distinct advantage over nanoparticle films due to their vertically aligned one-dimensional electron transport pathways, reduced grain-boundary scattering, and improved charge collection efficiency. However, the wide bandgap of TiO_2_ and interfacial recombination losses limit device performance. Nitrogen doping has therefore emerged as an effective strategy to modulate the electronic structure and enhance visible-light response.^94^

Nitrogen incorporation into TNTs-*via* annealing in NH_3_, urea-assisted treatments, sol–gel routes, or plasma-based processes-introduces localized states near the valence band, narrows the effective bandgap, and can improve charge-carrier separation when substitutional configurations dominate.^38,96,97^ As summarized in Table 9, multiple studies report increases in short-circuit current density (*J*_sc_), open-circuit voltage (*V*_oc_), and overall power conversion efficiency (PCE) upon nitrogen doping.

**Table 9:** Representative studies on N-TNT-based DSSCs

Study focus	Material form	Doping method	Device impact	Ref.	Stage (M/O/A)
Effect of N concentration on DSSC performance	Nanotube array	NH_3_ annealing	∼20% efficiency increase	94	O
N-doped TiO_2_ nanoparticles for DSSC photoanodes	Nanoparticles	Sol–gel (NH_4_NO_3_)	Enhanced *J*_sc_ and *V*_oc_	93	O
N-TNTs on Ti substrate	NT on Ti foil	Anodization + N doping	Moderate PCE increase	98	O
TNTs in DSSCs (review)	Various	Multiple	Visible-light response extension	38	M
N-doping impact in solid-state DSSCs	Solid-state device	Plasma doping	Improved charge transport	97	M
N-TNTs in DSSCs	Nanotubes	Annealing	Efficiency increase (4.7% → 7.3%)	99	O
Comparative doped TiO_2_ nanocrystals	Nanocrystals	Wet chemical synthesis	Comparative performance	100	M
N-doped TiO_2_ for solar H_2_ + DSSC synergy	Nanotubes	Urea-assisted doping	Enhanced H_2_ evolution + DSSC potential	101	O

For example, ammonia-annealed N-TNT arrays demonstrated efficiency improvements of ∼20% relative to undoped electrodes, primarily attributed to enhanced visible absorption and reduced recombination.^96^ Sol–gel-derived N-doped TiO_2_ nanoparticles used in DSSCs showed enhanced *J*_sc_ and *V*_oc_ values due to improved photoanode conductivity and dye loading efficiency.^100^ Similarly, anodized TNTs on Ti substrates followed by nitrogen doping exhibited moderate but reproducible increases in device efficiency, reflecting improved electron lifetime and interfacial transport.^102^

Importantly, the influence of nitrogen doping is strongly dependent on dopant configuration and concentration. Solid-state DSSC studies indicate that moderate nitrogen incorporation can facilitate interfacial charge transfer, whereas excessive mid-gap state formation may increase trap-assisted recombination.^97^ These findings underscore the necessity of controlled annealing severity and defect management, consistent with the defect-engineering principles discussed in Section 3.

Beyond single-dopant systems, co-doping and hybrid strategies have also been explored. Incorporating nitrogen alongside other non-metals or coupling TNTs with functional nanocrystals enables synergistic improvements in light harvesting and charge transport.^99,103^

Moreover, optimized N-TNT systems have demonstrated dual functionality in solar hydrogen evolution and dye degradation, suggesting broader applicability in integrated photoelectrochemical devices, including CO_2_ photoelectroreduction.^101,104^

Overall, the data compiled in Table 9 reveal a recurring trend: substitutional nitrogen incorporation combined with well-aligned nanotube morphology enhances photocurrent and device efficiency, whereas uncontrolled defect generation can offset these gains. Therefore, nitrogen doping should be regarded not as a simple bandgap modification strategy but as a targeted electronic-structure engineering approach tailored to device architecture and interfacial kinetics.^38,96,97,99,103^

##### 5.1.3.1. Critical assessment by research stage

The DSSC literature (Table 9) contains a relatively balanced mixture of stage-M and stage-O studies, because the solar-cell community routinely measures charge-transport and recombination lifetimes; nevertheless, the role of nitrogen is interpreted contradictorily, as a band-edge shift that improves injection in some reports and as a source of recombination centres in others. The framework of this review suggests that both are correct in different *µ*_o_ regimes—substitutional nitrogen with shallow vacancies improves injection, while over-reduction creates deep traps—but the annealing atmosphere, which decides between the two, is rarely reported. Stage-A work on module-scale N-TNT photoanodes is lacking.

#### 5.1.4 Fuel cells

TNTs have attracted attention in fuel-cell-related electrocatalysis owing to their semiconducting nature, high surface-to-volume ratio, and structural stability. Although pristine TiO_2_ exhibits limited intrinsic catalytic activity, its nanotubular architecture serves as an effective support for noble-metal nanoparticles, enabling enhanced dispersion, improved interfacial charge transfer, and increased electroactive surface area.

Hosseini *et al.*^102^ synthesized anodic TNT arrays and subsequently decorated them with electrochemically deposited Au nanoparticles derived from KAu(CN)_2_ precursors. The Au/TNT composite was evaluated for hydrazine oxidation, a reaction relevant to direct hydrazine fuel cells. Cyclic voltammetry measurements revealed significantly enhanced anodic current densities compared with bare Au electrodes, indicating that the nanotubular scaffold promotes improved catalytic utilization and mass transport.

In a subsequent study, Hosseini *et al.*^105^ reported Pt-decorated TNTs for galactose oxidation. The TiO_2_-Pt composite electrode demonstrated substantially higher current densities than both smooth Pt and unmodified TNTs under acidic and alkaline conditions. The performance enhancement was attributed to (i) increased active surface area, (ii) improved dispersion of Pt nanoparticles, and (iii) facilitated electron transfer across the TiO_2_-metal interface.

Overall, TNTs primarily function as structurally robust, high-area catalyst supports in fuel cell systems rather than as intrinsic electrocatalysts. Their role in stabilizing noble-metal nanoparticles and enhancing catalytic efficiency suggests potential for hybrid anodic architectures, particularly when defect engineering or heterostructure design is incorporated to improve conductivity and interfacial charge transport.

### 5.2 Fundamentals of photoelectrocatalysis in TiO_2_ nanotubes

Photocatalysis (PC) and photoelectrocatalysis (PEC) are both governed by the generation of electron–hole pairs upon semiconductor illumination with photons of energy equal to or exceeding the band gap. Upon light absorption, electrons are excited from the valence band (VB) to the conduction band (CB), leaving behind holes in the VB (eqn (4)). The redox capability of these photogenerated carriers is dictated by the relative energetic positions of the CB and VB with respect to the electrochemical potentials of target reactions. In TiO_2_-based systems, photogenerated holes possess strong oxidative potential and can oxidize surface-adsorbed water or hydroxide species to generate hydroxyl radicals (˙OH), which act as highly reactive oxidants (eqn (5)). Concurrently, conduction-band electrons can reduce dissolved oxygen to form superoxide radicals (O_2_˙^−^) (eqn (6)):^1,67,106^

4 Photocatalyst + *hv* → e^−^ + h^+^

5
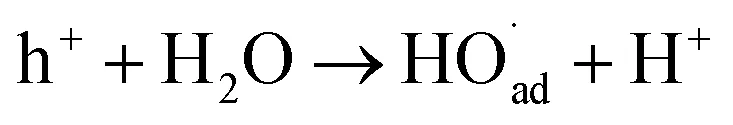

6 e^−^ + O_2_ → O_2_˙^−^

A major limitation of conventional photocatalysis is the rapid recombination of photogenerated carriers, which substantially reduces quantum efficiency and overall catalytic performance.^1,67,106^

PEC systems address this limitation by coupling illumination with an externally applied bias. In a typical PEC configuration, the semiconductor is deposited onto a conductive substrate to form a photoelectrode. The applied potential induces band bending at the semiconductor-electrolyte interface, facilitating spatial separation of electrons and holes and suppressing recombination losses.^107–110^

As illustrated schematically in Fig. 9, under illumination and positive bias (*E* > *E*_fb_ for n-type semiconductors), electrons are driven toward the counter electrode while holes accumulate at the semiconductor-electrolyte interface. This directional transport enhances oxidation kinetics at the photoanode surface and improves charge extraction efficiency. Consequently, PEC systems exhibit superior carrier utilization efficiency compared to standalone photocatalysis.^103^

**Fig. 9:**
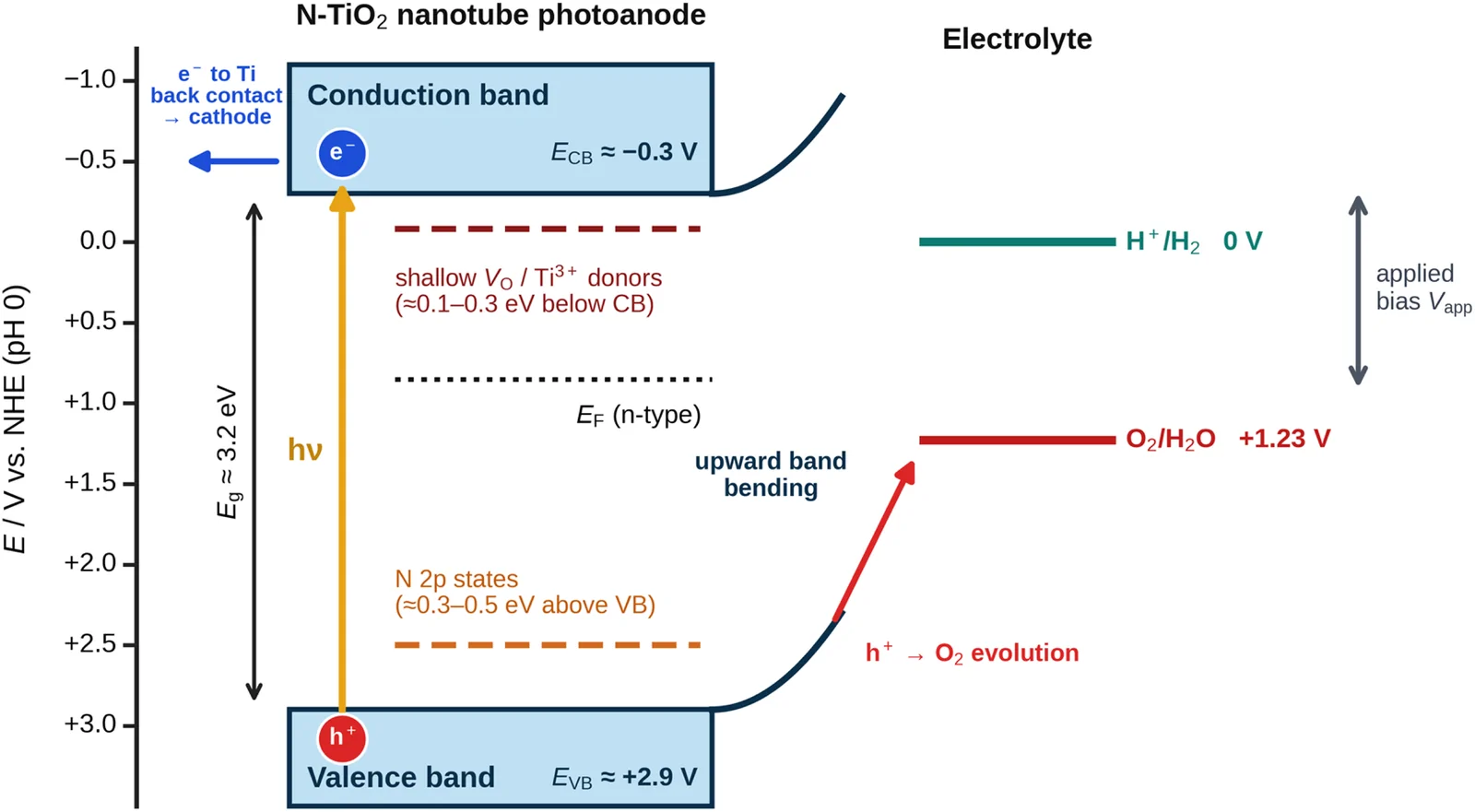
Energy-level diagram of an n-type N-doped TiO_2_ nanotube photoanode in a photoelectrochemical cell under illumination and applied bias, on the electrochemical scale (V *vs.* NHE, pH 0). The conduction- and valence-band edges of anatase (*E*_CB_ ≈ −0.3 V, *E*_VB_ ≈ +2.9 V; *E*_g_ ≈ 3.2 eV) are shown together with the H^+^/H_2_ (0 V) and O_2_/H_2_O (+1.23 V) redox levels, the Fermi level of the n-type semiconductor, the upward band bending at the semiconductor/electrolyte interface, and the applied bias *V*_app_. Shallow V_o_/Ti^3+^ donor levels (≈0.1–0.3 eV below the conduction band) and nitrogen-induced states (≈0.3–0.5 eV above the valence band) are indicated; photogenerated holes drive oxygen evolution at the interface while electrons are collected through the titanium back contact. Redrawn by the authors; band-edge and defect-level positions after ref. 31, 33 and 106.

#### 5.2.1. Critical assessment by research stage

Work on the fundamentals of photoelectrocatalysis in TNTs is, by definition, stage M, and it is here that the most quantitative defect data exist (Mott–Schottky donor densities, flat-band potentials, impedance spectra). The limitation is that these quantities are seldom connected to the nitrogen configuration or to the *µ*_o_ history of the sample, so that the observed donor densities cannot be assigned to V_o_, Ti^3+^ or nitrogen-related states. Reporting the N_sub_/N_int_ ratio together with N_D_, as the protocol of Section 3.7 requires, would convert this body of work into the mechanistic backbone the field currently lacks.

### 5.3 Photocatalytic and environmental applications

#### 5.3.1 Photocatalysis for organic pollutant degradation

Highly ordered TNT arrays produced *via* electrochemical anodization provide distinct advantages over conventional nanoparticulate photocatalysts due to their directional electron transport pathways, reduced grain-boundary scattering, and improved charge collection efficiency.^6,101^ These structural attributes make nanotubular architectures particularly suitable for photocatalytic degradation of organic pollutants under both UV and visible irradiation.^41^

Nitrogen doping modifies the electronic structure of TiO_2_ by introducing localized states near the valence band edge, frequently associated with Ti^3+^ centers and oxygen vacancies. This results in bandgap narrowing and enhanced visible-light absorption. However, photocatalytic efficiency depends strongly on nanotube geometry (length, wall thickness), nitrogen configuration (substitutional *vs.* interstitial), and dopant concentration.^21,22^

Mazierski *et al.* demonstrated that tuning anodization voltage (20–50 V) and duration (30–120 min) directly influenced nitrogen incorporation and nanotube geometry, thereby improving charge separation efficiency and hydroxyl radical (˙OH) generation during phenol degradation.^21^

Quantitative kinetic analyses further confirm the role of nitrogen doping. Le *et al.* reported that N-TNT arrays containing 9.47 at% nitrogen exhibited an apparent first-order degradation rate constant of *k*_app_ = 0.26 h^−1^ under visible light (*λ* ≥ 400 nm, 120 mW cm^−2^), representing a 125% enhancement relative to pristine nanotubes (*k*_app_ = 0.115 h^−1^).^41^

Co-modification strategies can further amplify performance. Ag/N co-modified TNTs achieved approximately 92% methylene blue degradation within 240 min under visible irradiation, with nearly nine-fold higher kinetic rates than undoped TiO_2_, highlighting the synergistic role of plasmonic enhancement and defect engineering.^111^

A broader comparison (summarized in Table 10) indicates that while N-TNTs significantly improve visible-light activity relative to pristine TiO_2_, their kinetic constants generally remain lower than those of advanced visible-light photocatalysts such as g-C_3_N_4_- and BiVO_4_-based heterostructures (*k*_app_ ≈ 0.03–0.07 min^−1^).^112^ Nevertheless, the structural robustness, stability, and facile fabrication of anodic nanotube arrays make N-TiO_2_ systems attractive candidates for hybrid photocatalytic architectures.

**Table 10:** Photocatalytic performance of various N-doped TiO_2_-based materials under visible light

Photocatalyst	Pollutant	Light source (*λ* nm)	Intensity	*k*_app_	Degradation (%)	System (PC/PEC)	Ref.	Stage (M/O/A)
Mo + N-TNT	MB	Visible	—	—	—	PC	113	O
N-TNT	Phenol	*λ* > 400 nm	—	—	96%	PC	21	O
Ag/N-TNT	MB	Visible	—	—	∼92%	PEC	63	O
Balanced N-TNT	RhB	*λ* > 420 nm	—	—	91%	PC	114	O
Plasma-doped N-TNT	MO	Visible	—	—	Enhanced (qual.)	PC		O
N-TNT (9.47 at%)	MB	*λ* ≥ 400 nm	120 mW cm^−2^	0.26 h^−1^	—	PC	22	O
g-C_3_N_4_/N-TNT composite	RhB	Visible	—	—	Strong enhancement	PC	40	O
Mo + N-TNT	MB	Visible	—	—	—	PC	55	O

Collectively, these findings suggest that optimal photocatalytic performance arises from coordinated control of nanotube geometry, nitrogen configuration, and defect density rather than from nitrogen concentration alone.^24,43^

##### 5.3.1.1. Critical assessment by research stage

Photocatalytic degradation studies (Table 10) are overwhelmingly stage O and are the least comparable body of work in this review: light sources, intensities, pollutant concentrations and catalyst loadings differ widely, apparent rate constants are often not normalised to irradiated area or catalyst mass, and the ‘degradation %’ metric depends on the reaction time chosen. The contradictory interpretation of nitrogen is most acute here—band-gap narrowing, vacancy-mediated visible absorption and nitrogen-induced recombination are all invoked—and the absence of defect quantification means that none can be excluded. Stage-A studies on real effluents, catalyst recovery and long-term visible-light stability are rare.

#### 5.3.2 Water splitting for hydrogen generation

Hydrogen generation through PEC water splitting is widely regarded as one of the most promising routes for sustainable solar-to-fuel conversion.^103,115,116^

For thermodynamically feasible overall water splitting, the conduction band minimum (CBM) of the semiconductor must lie above (more negative than) 0 V *vs.* NHE for proton reduction, while the valence band maximum (VBM) must be positioned below (more positive than) 1.23 V *vs.* NHE for oxygen evolution.^1^

TiO_2_ satisfies these energetic requirements and remains one of the most extensively studied photoanodes due to its chemical stability, non-toxicity, and suitable band alignment.^107^ Upon illumination, electrons are promoted from the valence band to the conduction band (eqn (7)), enabling proton reduction to hydrogen (eqn (8)), while holes oxidize water to oxygen (eqn (9)):

7 TiO_2_ + *hv* → e^−^ + h^+^

8 2H^+^ + 2e^−^ → H_2_

9 2H_2_O + 4h^+^ → O_2_ + 4H^+^

10 Overall reaction: 2H_2_O → O_2_ + 2H_2_

Despite these advantages, pristine TiO_2_ suffers from limited visible-light absorption and rapid carrier recombination. In PEC configurations under applied bias (Fig. 10), band bending at the semiconductor-electrolyte interface promotes directional charge separation, driving electrons toward the counter electrode and retaining holes at the photoanode surface, thereby enhancing photocurrent stability and hydrogen evolution rates.^117,118^

**Fig. 10:**
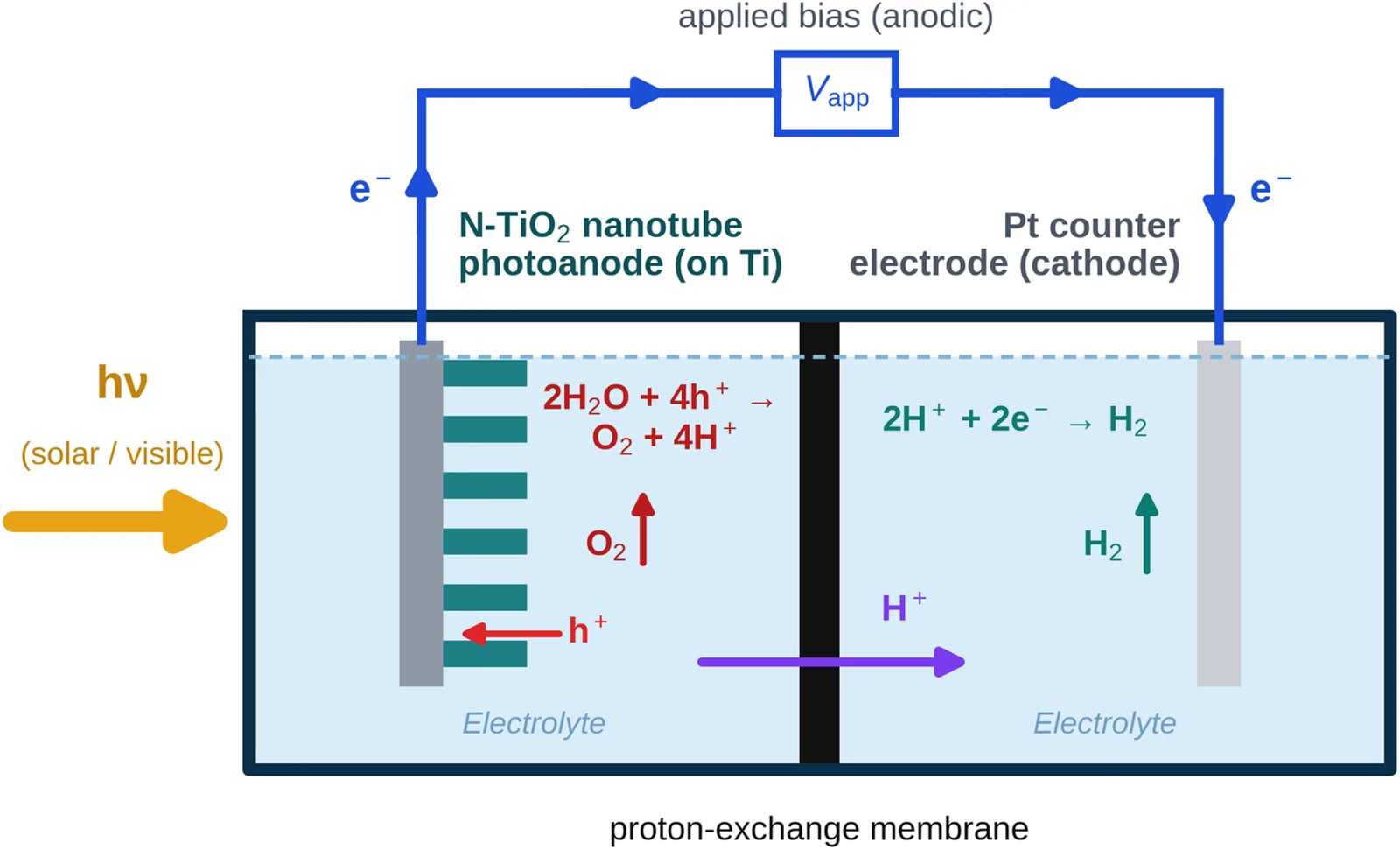
Schematic of a two-compartment photoelectrocatalytic water-splitting cell with an N-TiO_2_ nanotube photoanode on a Ti substrate and a Pt counter electrode separated by a proton-exchange membrane. Under illumination and an anodic applied bias *V*_app_, photogenerated holes oxidise water to O_2_ at the photoanode, protons cross the membrane, and electrons travel through the external circuit to reduce protons to H_2_ at the cathode. Redrawn by the authors after ref. 118.

Nitrogen doping modifies the electronic structure of TiO_2_ by introducing N 2p states near the valence band edge, effectively narrowing the band gap and extending absorption into the visible region. Quantitative studies demonstrate that N-doped TiO_2_ thin films achieved hydrogen evolution rates of approximately 601 µmol g^−1^ h^−1^ under simulated solar/visible irradiation-significantly exceeding those of undoped TiO_2_ under identical conditions.^119,120^

Furthermore, crystallographic orientation and facet engineering synergistically enhance performance. Orientation-controlled N-TiO_2_ films showed surface-area-normalized hydrogen evolution rates increasing from ∼7.6 × 10^2^ to ∼4.5 × 10^3^ µmol m^−2^ h^−1^ under AM 1.5 illumination,^120^ highlighting the combined importance of defect engineering and structural control.

Although visible-light-responsive semiconductors such as g-C_3_N_4_ and BiVO_4_ often exhibit broader solar absorption windows,^118,121,122^ N-TNT arrays offer superior chemical stability, controllable morphology, and compatibility with scalable anodic fabrication. Consequently, their optimal deployment is likely in heterostructured or co-catalyst-assisted PEC architectures rather than as standalone visible-light photocatalysts.

##### 5.3.2.1 Critical assessment by research stage

Water-splitting work with N-TNT photoanodes combines stage-M photoelectrochemical characterisation with stage-O photocurrent optimisation, and it is the application in which *operando* defect measurements (Section 7.4) are most feasible because the electrode is already under bias and illumination. Photocurrent enhancements are attributed to nitrogen-induced visible absorption, to vacancy-enhanced conductivity or to improved band bending, usually without the measurements that would distinguish them; the reported onset potentials and photocurrents also depend strongly on the back-contact quality discussed in Section 6.1. Long-term photostability at operating bias—the stage-A question—is rarely reported beyond a few hours.

## 6. Device-level optimization and system integration

Although substantial progress has been achieved in tuning the defect chemistry and electronic structure of N-TNTs, translating these intrinsic material modifications into practical device performance requires comprehensive device-level optimization. In vertically aligned anodic TNT arrays, performance is governed not only by nitrogen incorporation and oxygen vacancy control but also by electrode architecture, interfacial charge-transfer kinetics, conductive integration, and mass-loading management.^5,6,10,59^

### 6.1 Electrode architecture and electrical connectivity

A key advantage of anodically grown TNTs lies in their direct growth on Ti substrates, enabling binder-free electrode configurations with minimized contact resistance and enhanced mechanical stability.^5,6^ Compared with conventional slurry-cast nanoparticle electrodes, vertically aligned nanotube arrays provide continuous electron pathways and reduced grain-boundary resistance, which are essential for high-rate lithium storage and rapid charge extraction.

However, nanotube geometry critically influences electrochemical performance. Tube length, wall thickness, and pore diameter determine ionic diffusion distance and electron percolation efficiency. Excessive tube elongation increases Li^+^ diffusion resistance and polarization losses, whereas overly thin walls compromise mechanical integrity during repeated lithiation–delithiation cycles.^29,62,123^ Previous mechanistic studies have demonstrated that optimized geometric windows are required to balance areal capacity and rate capability.^5,29^ Therefore, geometry control must be co-optimized with nitrogen doping strategies to maintain structural robustness and transport efficiency. Fig. 11 summarizes the hierarchical device-level framework through which intrinsic defect modulation in N-TNTs is translated into enhanced electrochemical and photoelectrochemical performance.

**Fig. 11:**
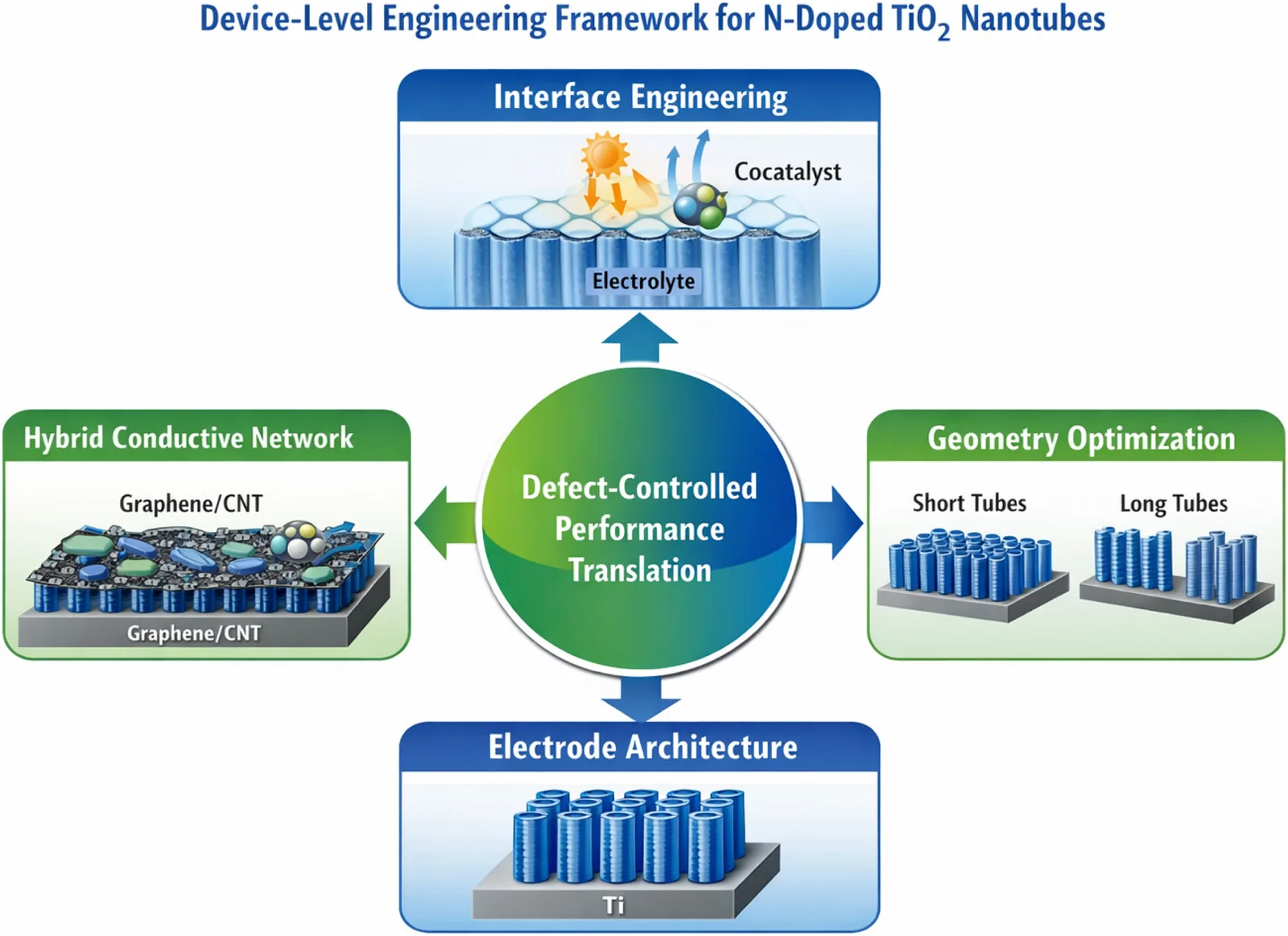
Schematic overview of device-level optimization strategies in N-TNTs linking defect engineering to practical performance.

How the nanotube array is wired into a device determines whether its intrinsic advantages are realised, and the electrode architecture differs markedly between applications (Fig. 12 and Table 11). In the most common configuration the N-TNT layer remains on the titanium foil or sheet from which it was grown, and the metallic titanium serves simultaneously as mechanical support and back current collector; this binder-free, self-supported geometry is used for lithium-ion anodes and supercapacitor electrodes, where the array is tested directly as the working electrode.^19,28–30^ For dye-sensitised solar cells and photoelectrochemical photoanodes that must be illuminated through the substrate, the array is instead grown on, or transferred to, transparent conducting oxide (FTO or ITO) glass, usually over a compact TiO_2_ under-layer that blocks recombination at the exposed conducting oxide.^38,94,98,99^ Free-standing membranes detached from the titanium substrate can be laminated onto copper, aluminium or nickel foils with a conductive silver-paste or carbon interlayer, which decouples the choice of current collector from the anodisation substrate; and for chemiresistive gas or photo sensors the array is contacted laterally through interdigitated noble-metal electrodes deposited on the tube tops.^8^

**Fig. 12:**
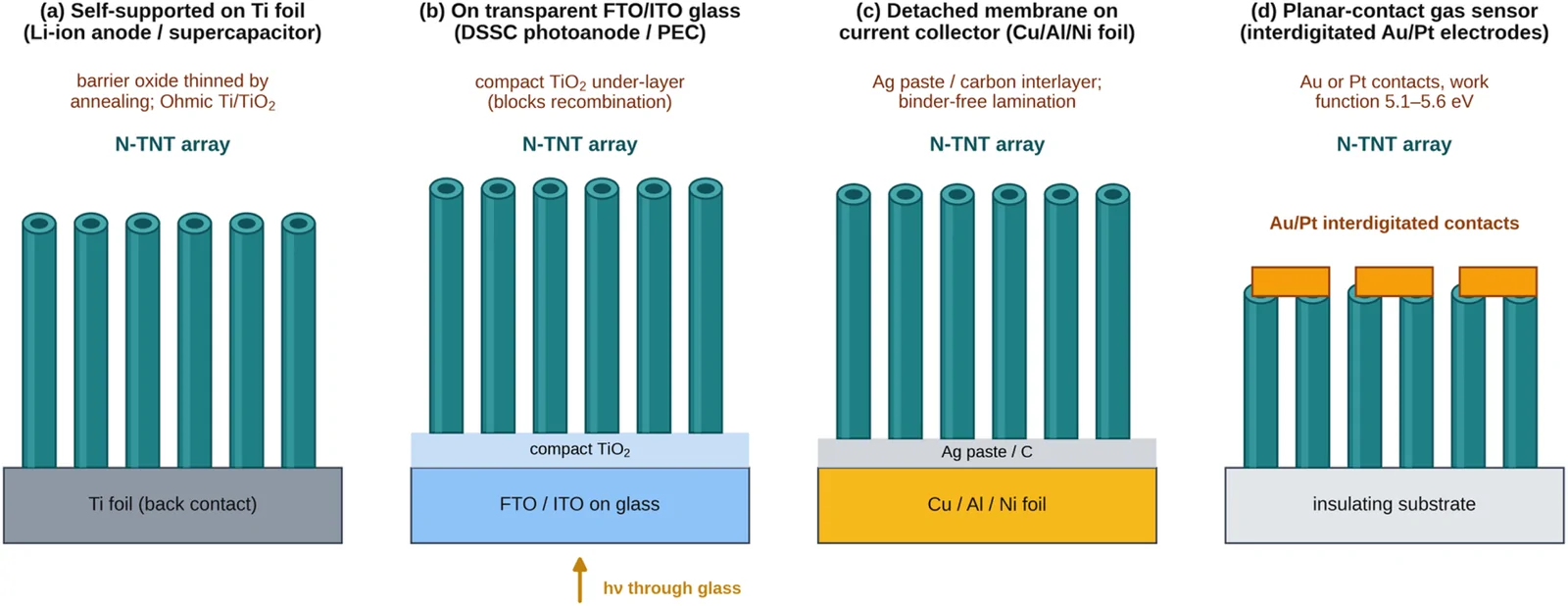
Electrode architectures used with N-TNT arrays in different applications. (a) Self-supported array on titanium foil, which acts as the back current collector (lithium-ion anodes, supercapacitors); the barrier oxide at the Ti/TiO_2_ interface is thinned and made ohmic by annealing. (b) Array on transparent conducting FTO/ITO glass with a compact TiO_2_ under-layer for front-side illumination (DSSC photoanodes, photoelectrochemical cells). (c) Detached membrane laminated onto a Cu, Al or Ni foil through a silver-paste or carbon interlayer. (d) Planar configuration with interdigitated Au or Pt contacts on an insulating substrate for chemiresistive gas or photo sensing.

**Table 11:** Electrode fabrication and contact engineering for N-TNT arrays by application

Application	Substrate/current collector	Contact metal and type	Contact formation and treatment	Practical notes	Ref.
Li-ion anode	Ti foil (self-supported array); Cu foil for detached membranes	Ti back contact—ohmic after anneal; Cu *via* Ag paste	Anneal 400–500 °C (reducing/N_2_/NH_3_) thins barrier oxide and raises donor density	Binder-free; report areal and gravimetric capacity; excessive tube length raises Li^+^ diffusion resistance	19, 29 and 30
Supercapacitor	Ti foil or Ti mesh; Ni/Al current collectors in assembled cells	Ti—ohmic; electrochemically reduced surface (Ti^3+^) lowers *R*_ct_ further	Anneal; optional electrochemical reduction or H_2_ treatment	Three- *vs.* two-electrode data not interchangeable; normalise per area and per mass	19 and 28
DSSC photoanode	FTO/ITO glass (front illumination) or Ti foil (back illumination)	FTO/ITO—ohmic to n-TiO_2_; Ag or Cu bus bars; Pt counter-electrode	TiCl_4_ treatment or compact TiO_2_ under-layer; sintering ≈450 °C; membrane transfer for front-side geometry	Back-side illumination through Ti foil is impossible—membrane transfer or FTO growth required	38, 94, 99 and 98
PEC photoanode	Ti foil (back contact) or FTO glass	Ti or Cu wire with Ag paste; epoxy-sealed junction	Anneal for ohmic back contact; edge insulation; define illuminated area	Report photocurrent per illuminated geometric area; state bias *vs.* reference electrode	106 and 118
Gas/photo sensor	Insulating substrate (planar) or Ti foil	Au or Pt interdigitated contacts (sputtered/evaporated); ohmic on defective n-TiO_2_, Schottky on stoichiometric surfaces	Contact anneal 300–450 °C; controlled *µ*_o_ of the array sets contact character	Contact type itself reflects the defect state; useful as a diagnostic	8

The quality of the electrical contact is governed by the barrier oxide that separates the tube bottoms from the titanium substrate. This compact layer, some tens of nanometres thick as formed, is amorphous and highly resistive and is the dominant series resistance of an as-anodised array. Post-anodisation annealing crystallises the barrier layer, thins it by partial dissolution of oxygen into the titanium, and—in a reducing or nitrogen-containing atmosphere—populates it with V_o_/Ti^3+^ donors, so that the Ti/TiO_2_ junction becomes ohmic.^5,6^ This is a further, often unappreciated, consequence of the *µ*_o_ set during post-treatment: the same reducing conditions that place the wall material in the synergy window also lower the contact resistance at the back interface. Electrochemical reduction of the array, which injects Ti^3+^ near the surface, acts in the same direction and is widely used to boost supercapacitor performance.^19^

Where a separate metal contact is applied, its work function relative to the electron affinity of n-type TiO_2_ decides between ohmic and rectifying behaviour. Low-work-function metals such as titanium and aluminium (≈4.3 eV) form ohmic contacts readily; high-work-function metals such as gold and platinum (≈5.1–5.6 eV) tend to form Schottky barriers on stoichiometric TiO_2_, but become ohmic on the heavily defective, V_o_-rich surfaces produced by reducing post-treatment, because the narrow depletion width permits tunnelling—a useful diagnostic of the defect state in its own right. Contact annealing (typically 300–450 °C) is used to interdiffuse the metal and the oxide and to remove interfacial adsorbates; silver paste or carbon interlayers provide compliant, low-resistance connections for membranes and for wire attachment; and epoxy sealing of the wire junction is essential in photoelectrochemical cells to define the illuminated area and exclude parasitic currents.^106,118^

Table 11 collects these practices for each application together with representative references. Two consequences follow for the interpretation of the literature. First, reported differences in rate capability, capacitance or photocurrent between N-TNT samples may originate at the back contact rather than in the wall material, so contact preparation belongs in the reporting protocol of Section 3.7 (as part of the *µ*_o_ proxy and the test conditions). Second, because contact resistance falls with the shallow-donor density, the same defect state that is optimal for the wall material is generally also optimal for the contact—a further reason why the synergy window of Fig. 13 is shared across applications.

**Fig. 13:**
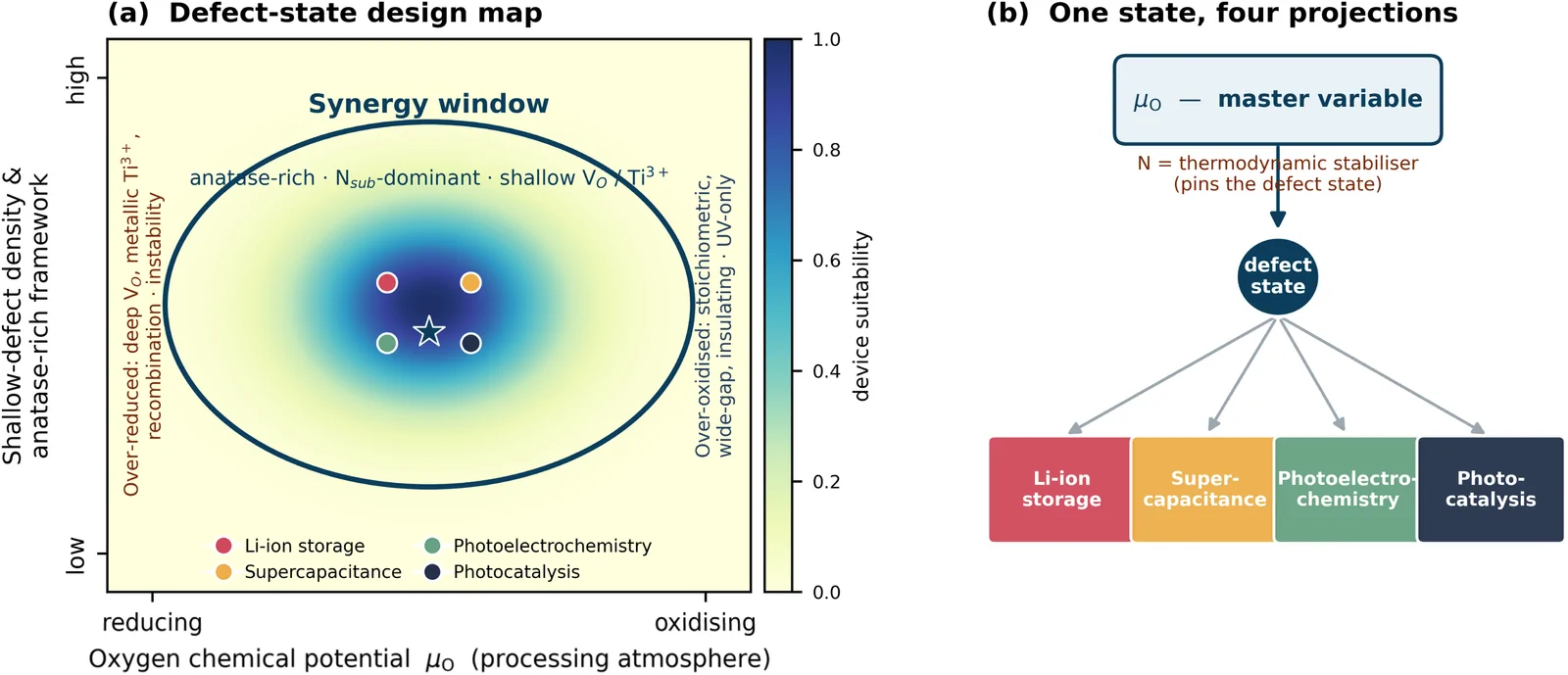
Defect-state design map for nitrogen-doped TiO_2_ nanotube arrays. (a) In the plane of oxygen chemical potential (*µ*_o_, set by the processing atmosphere) and shallow-defect density within an anatase-rich framework, device suitability peaks in a bounded synergy window; over-reduction promotes deep vacancies, recombination and instability, whereas over-oxidation restores a stoichiometric, wide-gap insulator. The optimal regimes for lithium-ion storage, supercapacitance, photoelectrochemistry and photocatalysis fall within the same window. (b) Because a single defect state—stabilised (pinned) by nitrogen and controlled by *µ*_o_—underlies all four functions, the applications are best viewed as projections of one defect equilibrium rather than as independent optimisation problems.

### 6.2 Charge-transfer engineering and interface control

Nitrogen incorporation modifies the electronic structure of TiO_2_ by introducing N 2p states and altering oxygen vacancy populations, thereby influencing carrier density and defect-state distribution.^12,15,31^ Moderate substitutional nitrogen doping has been reported to enhance electronic conductivity and facilitate Li^+^ insertion kinetics, reducing charge-transfer resistance (*R*_ct_) in battery systems.^19,29^ In contrast, excessive interstitial nitrogen and uncontrolled Ti^3+^/V_o_ formation generate deep trap states that act as recombination centers, increasing internal resistance and diminishing device stability.^12,31^

For PEC systems, interfacial band alignment between N-TNTs and the electrolyte is decisive for photovoltage generation and charge separation efficiency.^4,124^ Impedance spectroscopy analyses have revealed that optimized surface states and controlled defect densities reduce recombination losses and enhance interfacial charge-transfer kinetics.^52^ Surface passivation layers, cocatalyst deposition, and heterojunction formation have been shown to suppress surface recombination and improve photocurrent density under visible-light irradiation.^11,52,125^ Consequently, defect engineering must be integrated with interface optimization strategies to achieve measurable device-level improvements.

### 6.3 Hybrid architectures and conductive network integration

The intrinsically low electrical conductivity of TiO_2_ remains a limitation in high-power applications. To overcome this constraint, hybrid architectures incorporating conductive scaffolds such as graphene, carbon nanotubes, or conducting polymers have been extensively explored.^19,67^ In lithium-ion batteries and supercapacitors, N-TNT/graphene composites exhibit improved rate capability and cycling stability due to enhanced electron transport networks and mechanical buffering effects.^19^

Similarly, coupling N-TNTs with narrow-bandgap semiconductors (*e.g.*, g-C_3_N_4_ or CdS) forms heterojunction systems that promote spatial separation of photogenerated carriers and extend visible-light absorption.^11,22,126^ While such hybridization enhances device performance, it introduces additional complexity in band alignment, defect redistribution, and long-term stability. Therefore, hybrid design must be guided by controlled interface engineering rather than empirical compositional variation.

### 6.4 Mass loading, thickness effects, and practical trade-offs

A recurring limitation in vertically aligned nanotube arrays is the trade-off between increased active material loading and transport efficiency. Increasing nanotube length enhances areal capacity but simultaneously increases ion diffusion resistance and recombination probability.^5,62^ In lithium-ion systems, excessive thickness can lead to mechanical stress accumulation and capacity fading during extended cycling.^29^

In photoelectrochemical devices, thick nanotube layers reduce light penetration depth and increase bulk recombination, limiting incident photon-to-current efficiency (IPCE).^4,124^ Thus, practical device engineering requires identifying optimal geometric regimes that balance surface area, electronic conductivity, ionic transport, and structural durability.

To systematically summarize the key device-level factors governing the translation of defect engineering into measurable performance, Table 12 compiles the principal architectural, interfacial, and geometric parameters influencing N-TNT systems across different energy applications. The table highlights both performance-enhancing effects and associated trade-offs, emphasizing the importance of balanced optimization rather than isolated parameter tuning.

**Table 12:** Summary of key device-level parameters governing the performance of N-TNT architectures across energy applications

Device-level parameter	Device type	Performance enhancement	Limitation/trade-off	Key references
Direct growth on Ti (binder-free architecture)	LIB, supercapacitor	Reduced contact resistance; improved mechanical stability	Limited flexibility in substrate choice	5 and 6
Tube length optimization	LIB, PEC	Increased areal capacity; enhanced light absorption	Higher ion diffusion resistance at excessive length	4, 29 and 62
Wall thickness control	LIB	Improved structural robustness during cycling	Thin walls may lead to mechanical instability	29 and 62
Substitutional N doping	LIB, PEC	Enhanced conductivity; improved charge transfer	Excess doping may create recombination centers	12, 15 and 31
Oxygen vacancy modulation	LIB, PEC	Improved carrier density; reduced *R*_ct_	Deep traps if uncontrolled	12, 31 and 62
Graphene/CNT integration	LIB, supercapacitor	Enhanced electron transport; improved rate capability	Interface mismatch; scalability concerns	19 and 67
Heterojunction formation (*e.g.*, g-C_3_N_4_, CdS)	PEC, photocatalysis	Improved charge separation; extended visible-light response	Band misalignment risk	11, 22 and 52
Cocatalyst deposition	PEC	Reduced surface recombination; improved photocurrent	Added fabrication complexity	4 and 124
Increased nanotube thickness	LIB, PEC	Higher active mass loading	Reduced transport efficiency	4 and 29
Defect synergy window control	All energy devices	Balanced conductivity and stability	Narrow optimization range	12, 31 and 62

## 7. Stability, scalability and practical implementation challenges

Despite substantial progress in tailoring the defect chemistry and device-level performance of N-TNT arrays, several barriers still limit their transition from proof-of-concept demonstrations to reliable, scalable technologies. The most persistent issues include (i) stability under realistic electrochemical/photoelectrochemical operation, (ii) reproducibility of nitrogen configuration and defect populations, and (iii) industrial scalability of anodization and post-doping routes.^7,10,59^

### 7.1 Operational stability under electrochemical and photoelectrochemical conditions

In lithium-ion systems, long-term cycling can impose repeated mechanical and chemo-structural stresses on nanotube walls, potentially leading to gradual loss of electrical connectivity, microcrack formation, and polarization growth-effects that become more pronounced at high rates or high areal loadings. Thermal history is also critical: annealing-induced crystallization improves transport, yet high-temperature treatment can trigger morphology/phase evolution and support-related degradation that undermines nanotube integrity.^127^

For PEC operation, stability is further challenged by surface recombination, defect oxidation, and interfacial chemistry. In particular, nitrogen-related states and associated Ti^3+^/oxygen-vacancy (V_o_) populations can evolve under oxidative bias and illumination, causing drift in band energetics and reduced visible-light activity over time.^128,129^ Therefore, stability assessment should move beyond initial photocurrent/capacity values and include durability metrics under realistic illumination/electrolyte/bias protocols.

### 7.2 Reproducibility of defect chemistry and nitrogen configuration

A recurring limitation across the N-TNT literature is that performance cannot be correlated to “nitrogen content” alone; rather, the configuration (substitutional *vs.* interstitial) and the coupled defect equilibrium (Ti^3+^/V_o_*vs.* deep traps) govern transport and recombination.^33,130^ Even when nominally similar treatments are used, variations in local nitridation depth, atmosphere purity, and annealing profiles can yield non-uniform dopant distributions and inconsistent device outcomes.^10,59^

Moreover, photocatalysis/PEC performance is highly sensitive to surface chemistry and defect-mediated trapping processes. Mechanistic reviews emphasize that uncontrolled defect overpopulation increases recombination losses, while a controlled defect regime is required to enhance charge separation and interfacial kinetics.^128,129^ Consequently, standardized reporting (dopant quantification, defect spectroscopy, and comparable device test conditions) is essential for reproducible structure–performance mapping.

### 7.3 Scalability of synthesis and post-doping routes

Anodization itself is relatively scalable, but maintaining large-area uniformity (tube diameter/length distribution) and consistent post-treatment outcomes remains challenging at industrial throughput.^10,59^ Post-doping strategies such as high-temperature ammonia annealing, plasma treatments, or ion implantation can be effective but may raise cost, safety, and uniformity concerns for large substrates.^10^ Comprehensive process-level reviews highlight that electrolyte composition, applied potential, and heat treatment strongly affect crystallinity and defect formation, implying that scale-up must preserve tight process control to avoid batch-to-batch variability.^7,10,59^

### 7.4 *Operando* and *in situ* probes of the defect equilibrium in N-TNTs

The framework of this review makes a prediction that *ex situ* measurements cannot test: that the shallow-V_o_/Ti^3+^ population, and hence the position of a sample within the synergy window, is a dynamic quantity that responds to bias, illumination, electrolyte and temperature during operation, and that its drift is the microscopic origin of the degradation phenomena discussed in Section 7.1. Testing this requires probes that follow the defect state *in situ* or *operando*. Table 13 summarises the techniques that have been applied to N-TNTs and closely related TiO_2_ systems, the component of the defect state each probes, and their respective advantages and limitations.

**Table 13:** *In situ* and *operando* techniques applicable to the defect equilibrium in N-TNT systems: what each probes, the conditions accessible, and their principal advantages and limitations

Technique	Component of the defect state probed	Accessible conditions	Advantages	Limitations	Ref.
*In situ*/*operando* XAS (Ti K-edge XANES/EXAFS) and X-ray spectromicroscopy	Ti oxidation state (Ti^3+^), local coordination, anatase/rutile fingerprint; spatial distribution along tubes	Temperature, gas atmosphere; electrolyte and bias with dedicated cells	Element-specific, bulk-sensitive, quantitative; phase and chemical-state mapping	Synchrotron access; volume averaging; beam-induced reduction	131
*In situ* EPR (under illumination)	Paramagnetic Ti^3+^; N-centred radicals; superoxide/trapped holes	Illumination, gas atmosphere; usually low temperature	Highest sensitivity to defects; identifies photoactive species directly	Quantification difficult; limited operation under electrochemical bias	15
*In situ*/near-ambient-pressure XPS	Ti 2p (Ti^3+^/Ti^4+^), N 1s (N_sub_/N_int_), O 1s (V_o_-related, OH)	Gas/vapour, illumination; limited electrolyte	Direct speciation of N and Ti; basis of the reporting protocol	Surface sensitivity (few nm); charging; UHV/NAP constraints	40 and 72
*In situ* Raman (temperature-dependent)	Anatase *E*_g_ mode shift/broadening (V_o_, N); anatase → rutile transition	Temperature, gas, some electrolytes	Fast, laboratory-based, phase-sensitive	Indirect for V_o_ quantification; fluorescence; laser heating	72
*In situ* XRD/TEM heating	Crystallisation, phase transformation, grain growth, wall integrity	Temperature, gas	Direct structural evolution; TEM gives tube-level resolution	No direct defect chemistry; TEM beam can reduce TiO_2_	27
PEIS/Mott–Schottky under illumination	Donor density N_D_ (∝ shallow V_o_), flat-band potential, *R*_ct_	Bias, illumination, electrolyte; repeatable during stability tests	*Operando* by construction; quantitative N_D_; laboratory-based	Model-dependent for tube geometry; frequency dispersion must be reported	106
*Operando* UV-vis/DRS, spectroelectrochemistry	Ti^3+^-related visible/NIR absorption; band-gap shift	Bias, illumination	Simple; follows defect population in real time	Qualitative; overlapping absorptions; not yet reported *operando* for N-TNTs	Proposed

X-ray absorption spectroscopy at the Ti K-edge, and the associated X-ray spectromicroscopy, is the most direct bulk-sensitive probe of the titanium oxidation state and local coordination; it has been used to follow the anatase-to-rutile transition of anodic TiO_2_ nanotubes as a function of annealing and to resolve the phase and chemical-state distribution along individual tubes.^131^ With electrochemical or photoelectrochemical cells it can, in principle, follow Ti^3+^ formation under bias, but the synchrotron requirement, the averaging over the probed volume and the risk of beam-induced reduction limit its routine use. Electron paramagnetic resonance is the most sensitive probe of paramagnetic Ti^3+^ and of the nitrogen-centred radicals that carry the visible-light photoactivity; *in situ* EPR under illumination established the identity of the photoactive nitrogen species and their coupling to oxygen vacancies,^15^ although quantification and operation under electrochemical bias remain difficult and measurements are often performed at low temperature.

X-ray photoelectron spectroscopy provides the speciation on which the reporting protocol of Section 3.7 relies—the Ti 2p (Ti^3+^/Ti^4+^), N 1s (N_sub_/N_int_) and O 1s (lattice oxygen, vacancy-related and hydroxyl components) signatures^40,72^—and its near-ambient-pressure variant extends this to gas and vapour environments and to illumination; its surface sensitivity of a few nanometres is an advantage for plasma-treated arrays and a limitation for bulk-nitrided ones. Raman spectroscopy is the most accessible *in situ* probe of the anatase *E*_g_ mode, whose shift and broadening report on V_o_ and nitrogen incorporation and whose disappearance marks the anatase → rutile transition;^72^ it is fast and lab-based but indirect for vacancy quantification. *In situ* XRD and TEM heating experiments follow crystallisation, phase transformation and grain growth directly^27^ but do not access the defect chemistry itself, and electron-beam effects in TEM can reduce TiO_2_.

The electrochemical probes are *operando* by construction. Photoelectrochemical impedance spectroscopy and Mott–Schottky analysis under illumination yield the donor density N_D_—the electrical counterpart of the shallow-V_o_ density—together with the flat-band potential and the charge-transfer resistance, and can be repeated throughout a stability test;^106^ their interpretation is model-dependent for nanotube geometries, and the frequency dispersion of the capacitance must be reported. *Operando* UV-vis or diffuse-reflectance spectroscopy and spectroelectrochemistry follow the defect-related visible/near-infrared absorption associated with Ti^3+^ and the band-gap shift under bias, and are simple to implement, but overlapping absorptions make them qualitative.

The critical conclusion of this survey is that, in the large majority of N-TNT studies, the defect state is characterised only *ex situ*, before and occasionally after testing. The evolution of the V_o_/Ti^3+^ equilibrium during cycling, under sustained illumination or under bias—and therefore whether the synergy window is stationary or drifts, and whether nitrogen indeed pins it—remains essentially unmeasured for nanotube arrays. Combining an *operando* electrical probe (PEIS/Mott–Schottky) with one *operando* spectroscopic probe (XAS or EPR) on the same electrode would be sufficient to test the central hypothesis of this review, and constitutes the first priority of the roadmap set out in the conclusions.

### 7.5 Practical outlook

Overall, bridging the gap between laboratory-scale demonstrations and real devices requires a shift toward *operando*/long-term testing, defect-quantification workflows, and scalable processing windows that preserve both nanotube morphology and defect stability.^7,10,59,129^ A robust engineering roadmap should therefore integrate durability-focused metrics (capacity retention/photocurrent retention, impedance growth, and structural integrity) with standardized reporting of dopant configuration and defect populations.

To consolidate the principal barriers limiting the practical implementation of N-TNT systems, Table 14 summarizes the dominant stability, reproducibility, and scalability challenges, their underlying causes, device-level consequences, and potential mitigation strategies. This structured comparison emphasizes that long-term durability and process standardization are as critical as intrinsic defect optimization.

**Table 14:** Key stability and scalability challenges affecting N-TNT energy devices and their mitigation strategies

Challenge	Root cause	Impact on device performance	Mitigation strategy	Key references
Nitrogen dopant instability	Dopant oxidation or redistribution under bias/thermal stress	Performance decay; reduced visible-light activity	Surface passivation; controlled annealing; *operando* monitoring	33
Excess oxygen vacancies (V_o_ overpopulation)	Uncontrolled reduction or aggressive nitridation	Deep trap formation; increased recombination; higher *R*_ct_	Optimized defect window; moderate annealing atmosphere control	33, 128 and 129
Nanotube wall degradation during cycling	Mechanical stress from lithiation–delithiation	Capacity fading; structural cracking	Optimized wall thickness; moderate tube length design	29 and 62
Large-area uniformity issues	Anodization parameter fluctuation	Batch-to-batch variability; inconsistent device metrics	Process standardization; electrolyte and voltage control	7, 10 and 59
Scalability of nitridation routes	High-temperature NH_3_ annealing; plasma cost; safety constraints	Increased fabrication cost; industrial limitations	Low-temperature plasma; solution-based doping; roll-to-roll optimization	10 and 59
Long-term photoelectrochemical stability	Surface recombination; photocorrosion	Photocurrent decline; reduced IPCE	Cocatalyst deposition; heterojunction stabilization	3, 124 and 129

## 8. Conclusions and perspectives

The nitrogen-doped TiO_2_ nanotube literature is often read as a search for the right nitrogen chemistry. The evidence assembled in this review supports a sharper, and we believe more useful, claim: nitrogen is not the active ingredient—it is the stabiliser. Across lithium-ion storage, supercapacitance, photoelectrochemical water splitting and visible-light photocatalysis, the property that consistently tracks device performance is the coupled population of shallow oxygen vacancies and Ti^3+^ centres near the nanotube wall, not the absolute nitrogen concentration. Nitrogen matters because substitutional incorporation lowers the formation energy of these shallow donors and pins them against re-oxidation, thereby widening the processing window in which a favourable defect state can be reached and retained. The variable the experimentalist truly controls is therefore the oxygen chemical potential, *µ*_o_, imposed through anodization chemistry, annealing atmosphere and temperature, and the nitridation route. Recognising *µ*_o_ as the master variable reorganises a fragmented body of work into a single, predictive picture.

The most immediate consequence is that the four applications are projections of one defect state. When performance data are re-plotted against the underlying defect configuration rather than against nitrogen loading, their apparently disparate optima converge. Fig. 13 renders this as a defect-state design map in the *µ*_o_-defect-density plane: strongly reducing conditions over-populate deep vacancies and metallic Ti^3+^, promoting recombination and structural instability, whereas strongly oxidising conditions restore stoichiometry and revert the material to a wide-gap, poorly conducting oxide. Between these limits lies a bounded synergy window—anatase-rich with a controlled rutile fraction, substitutional-nitrogen-dominant, and carrying a moderate density of shallow (not deep) vacancies—in which visible-light absorption, donor density, interfacial charge-transfer kinetics and framework stability are simultaneously favourable. The same window underlies the best reported lithium-storage, supercapacitive, photoelectrochemical and photocatalytic devices; they differ mainly in which projection of the window is being read out.

To make this actionable we propose a compact, transferable defect-state descriptor built from three routinely measurable quantities: (i) the substitutional-to-interstitial nitrogen ratio, N_sub_/N_int_, from deconvolved N 1s X-ray photoelectron spectra (≈396–397 eV *versus* ≈399–400 eV); (ii) the density of shallow oxygen vacancies and Ti^3+^ centres, from electron paramagnetic resonance and valence-band/O 1s XPS; and (iii) the anatase mass fraction with its controlled rutile content, from Rietveld-refined XRD. We argue that device performance across all four applications is maximised within a bounded region of this three-dimensional descriptor space—the quantitative embodiment of the synergy window—and that reporting where a material sits in this space is far more predictive than reporting its nitrogen content alone. This descriptor is deliberately model-light and can be computed from data most laboratories already collect.

Realising a predictive field, however, requires comparability that the current literature lacks. We therefore call for a minimum defect-reporting protocol to accompany N-TNT performance claims: Rietveld-refined phase fractions and crystallite size; quantified N 1s speciation; an explicit measure of shallow-vacancy/Ti^3+^ density; nanotube geometry (diameter, wall thickness, length and porosity); and a stated *µ*_o_ proxy (annealing pO_2_, temperature and time, together with the nitridation conditions). Performance should be normalised both per geometric area and per active mass. Adopting even a subset of this protocol would allow results from different laboratories to be placed on the same design map and would convert isolated record values into cumulative, comparable knowledge. The protocol is set out in full in Section 3.7 (Fig. 3 and Table 3).

Looking further ahead, two developments would turn this framework from a descriptive lens into an engineering discipline. The first is *operando* defect metrology—spectroscopic and electrochemical probes of shallow vacancies and Ti^3+^ under working bias, illumination and cycling—which would test directly whether the synergy window is stationary or drifts during operation, and thereby link defect dynamics to degradation. The second is closed-loop, machine-learning-guided synthesis, in which *µ*_o_-defining process parameters are the control variables, the defect-state descriptor is the measured state, and device metrics are the objective. Because *µ*_o_ is a small, physically meaningful set of knobs, N-TNT arrays are an unusually tractable testbed for autonomous, defect-targeted materials optimisation. The *operando* techniques available for this purpose, and their limitations, are surveyed in Section 7.4 (Table 13).

Framing nitrogen-doped TiO_2_ nanotubes as a single defect equilibrium controlled by the oxygen chemical potential does more than tidy an active subfield. It replaces application-specific trial and error with one transferable design principle: set the defect state, and the functions follow. We expect the same logic—identify the master thermodynamic variable, treat the diverse functionalities as projections of one defect equilibrium, and report against a shared descriptor—to apply broadly to defective oxide nanomaterials, from doped ZnO and WO_3_ to the growing family of engineered high-defect-density semiconductors. If adopted, this reframing offers a route to N-TNT devices, and to defective-oxide devices more generally, that are designed rather than discovered.

## Author contributions

All authors contributed to the conception, design and scope of the review, the analysis of the published literature, and the preparation of the manuscript. conceptualization, methodology, design of the review framework: Hakimeh Ghazaie, Shahab Khameneh Asl, Shahin Khameneh Asl. Data curation, compilation and verification of the literature data: Hakimeh Ghazaie, Shahab Khameneh Asl, Shahin Khameneh Asl, Alisher Kahramonov, Utkirjon Sharopov. Formal analysis, software, validation: Nurbek Rakhimov, Shohruh Sayfulloev. Investigation, resources: Hakimeh Ghazaie, Sardorbek Saydaliev. writing – original draft: Hakimeh Ghazaie, Nurbek Rakhimov, Shohruh Sayfulloev. Writing – review & editing: Hakimeh Ghazaie, Utkirjon Sharopov, Sardorbek Saydaliev. All authors read and approved the final manuscript.

## Conflicts of interest

There are no conflicts of interest to declare. The authors alone are responsible for the content and writing of this article.

## Abbreviations

TNT: TiO_2_ nanotube (array)
N-TNT: Nitrogen-doped TiO_2_ nanotube
N_sub_: Substitutional nitrogen (replacing lattice oxygen)
N_int_: Interstitial nitrogen
V_o_: Oxygen vacancy
Ti^3+^: Reduced titanium centre
*µ*_o_
: Oxygen chemical potential (set by processing atmosphere, temperature and time)
pO_2_: Oxygen partial pressure
ECB/EVB: Conduction-/valence-band edge
EF: Fermi level
*E*_g_
: Band gap
N_D_: Donor density
*D*, *t*, *L*: Nanotube diameter, wall thickness and length
LIB: Lithium-ion battery
SC: Supercapacitor
EDLC: Electric double-layer capacitor
DSSC: Dye-sensitized solar cell
PEC: Photoelectrochemical (cell)
PC: Photocatalysis
XRD: X-ray diffraction
XPS: X-ray photoelectron spectroscopy
VB-XPS: Valence-band XPS
EPR: Electron paramagnetic resonance
XAS: X-ray absorption spectroscopy
NAP-XPS: Near-ambient-pressure XPS
PEIS: Photoelectrochemical impedance spectroscopy
DFT: Density functional theory
DRS: Diffuse-reflectance spectroscopy
rGO: Reduced graphene oxide
FTO/ITO: Fluorine-/indium-doped tin oxide
CRediT: Contributor roles taxonomy stages of research used in Tables 7–10
M: Mechanism exploration
O: Performance optimisation
A: Practical application

## Data Availability

No primary research data were generated for this review. All data discussed are taken from the publications cited in the reference list and are available from those sources; the datasets compiled in the tables of this review are available from the corresponding author upon reasonable request.
